# First-Line treatment for transplant-ineligible newly diagnosed multiple myeloma: a Bayesian network meta-analysis of efficacy, safety, and high-risk subgroups

**DOI:** 10.3389/fimmu.2026.1869797

**Published:** 2026-08-24

**Authors:** Dani Zhong, Hao Cheng, Lin Zeng, Chun Tian, Chunchun Liang, Hongyu Wei, Mengling Xu, Meili Pang, Qing Ke, Xiaohong Tan, Hong Cen, Chengcheng Liao

**Affiliations:** 1 Department of Hematology/Oncology, Guangxi Medical University Cancer Hospital, Nanning, Guangxi, China; 2 Department of Thyroid and Breast Surgery, The First Affiliated Hospital of Guangxi University of Chinese Medicine, Nanning, Guangxi, China; 3 State Key Laboratory of Targeting Oncology, Guangxi Medical University, Nanning, Guangxi, China

**Keywords:** anti-CD38, daratumumab, immunotherapy, isatuximab, multiple myeloma, network meta-analysis, transplant-ineligible

## Abstract

Anti-CD38 monoclonal antibody-based regimens have emerged as the first-line standard of care for transplant-ineligible newly diagnosed multiple myeloma (NDMM), yet direct head-to-head comparisons between individual regimens remain limited. The systematic review identified 11 eligible randomised controlled trials; 6 of these, involving 3162 patients, entered the Bayesian network meta-analysis to systematically evaluate the efficacy, safety, and benefits in the cytogenetically high-risk subgroup across 7 treatment nodes, keeping the dexamethasone-sparing IFM2017–03 schedule (D-R–limited dexamethasone) separate from continuous D-Rd. All four anti-CD38-containing regimens improved progression-free survival (PFS) relative to lenalidomide–dexamethasone, with D-VRd ranking highest (SUCRA 81.5%). For overall survival (OS), D-R–limited dexamethasone ranked highest (SUCRA 87.4%), but OS follow-up is considerably less mature for the quadruplet trials, and no pairwise comparison among the anti-CD38-containing regimens reached significance for either outcome. High-risk subgroup estimates could not rank regimens against one another, but both daratumumab–lenalidomide regimens retained a progression-free survival benefit relative to lenalidomide–dexamethasone. Regarding safety, several anti-CD38-containing regimens increased grade ≥3 neutropenia, whereas infection signals were regimen- and endpoint-specific. This study provides comprehensive evidence to inform individualized first-line treatment decisions for transplant-ineligible NDMM patients.

## 1 Introduction

Multiple myeloma (MM) is a malignant neoplasm of terminally differentiated plasma cells and the second most common haematological malignancy after non-Hodgkin lymphoma (1), leading to complications including renal failure, bone disease, and anaemia (2, 3). Globally, MM accounts for approximately 1% of all cancers and 10% of haematological malignancies, posing a substantial health burden (4). According to GLOBOCAN 2022 data, MM accounted for 0.9% of new cancer cases worldwide (187,774 cases) and 1.2% of cancer deaths (121,252 deaths), ranking 21st for incidence and 17th for mortality globally, with higher incidence rates in countries with high Human Development Index (HDI) and marked prognostic variation according to risk stratification and population differences (5, 6). The majority of MM patients are aged 70 years or older (7), and although novel agents and autologous haematopoietic stem cell transplantation (ASCT) have significantly improved survival for some patients, a considerable proportion remain ineligible for transplantation due to advanced age, comorbidities, or poor performance status (8). Therefore, exploring more effective treatment strategies for this large population with significant unmet medical needs is of substantial clinical importance.

Over the past decade, the widespread use of proteasome inhibitors and immunomodulatory drugs has significantly improved survival outcomes for MM patients (9). Lenalidomide plus dexamethasone (Rd) and bortezomib plus lenalidomide and dexamethasone (VRd) have long served as core first-line regimens for transplant-ineligible newly diagnosed patients (10). In recent years, the introduction of anti-CD38 monoclonal antibodies, represented by daratumumab and isatuximab, has further revolutionised the treatment landscape (10, 11). The MAIA trial demonstrated that daratumumab plus Rd (D-Rd) significantly prolonged both progression-free survival (PFS) and overall survival (OS) compared with Rd alone (HR 0.55 and 0.66, respectively) (12, 13); the CEPHEUS and IMROZ trials established the PFS superiority of daratumumab plus VRd (D-VRd) and isatuximab plus VRd (Isa-VRd) over VRd, respectively (14, 15) and the ENDURANCE trial compared carfilzomib plus Rd (KRd) with VRd (16). Clinicians now have a broader and more effective array of treatment options for their patients.

Despite the expanding treatment landscape, with D-VRd, D-Rd, Isa-VRd, KRd, VRd, and Rd all recommended by clinical guidelines (10), head-to-head trials directly comparing these regimens are lacking, precluding direct comparison of their efficacy and safety (17). Conventional meta-analyses can only perform limited pairwise comparisons and cannot comprehensively synthesise evidence across all regimens, posing challenges for clinicians selecting the optimal regimen for transplant-ineligible patients. Network meta-analysis (NMA) integrates both direct and indirect evidence, enabling quantification of relative benefits among multiple regimens in the absence of head-to-head trials, thereby providing more comprehensive evidence for clinical decision-making (18, 19). Accordingly, this study employed Bayesian NMA to systematically evaluate and compare the efficacy (PFS, OS) and safety of D-R–limited dexamethasone, D-Rd, D-VRd, Isa-VRd, KRd, VRd, and Rd in transplant-ineligible NDMM patients, with treatment ranking to clarify the relative merits of each regimen and provide evidence-based guidance for first-line treatment decisions.

## 2 Methods

### 2.1 Search strategy

**Figure 1:**
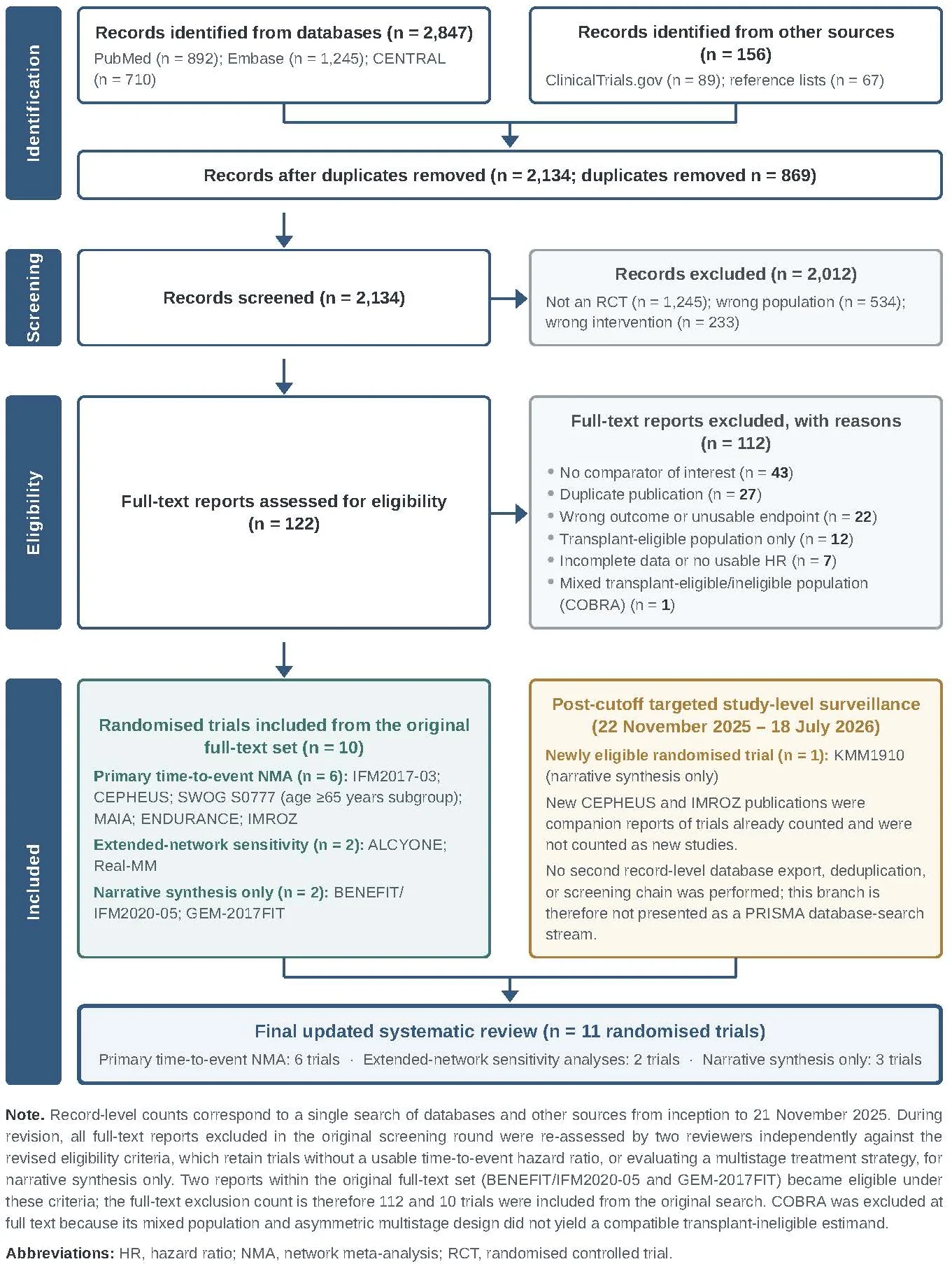
PRISMA flowchart of study screening process.

We systematically searched PubMed, Embase, and the Cochrane Central Register of Controlled Trials (CENTRAL) from inception to November 21, 2025, using a combination of Medical Subject Headings (MeSH) terms and free-text keywords related to disease (multiple myeloma, etc.), population (newly diagnosed, transplant-ineligible, etc.), interventions (daratumumab, lenalidomide, etc.), and study design (randomised controlled trial, RCT, etc.). Supplementary searches included ClinicalTrials.gov, the World Health Organization (WHO) International Clinical Trials Registry Platform (ICTRP), conference abstracts from the American Society of Hematology (ASH), European Hematology Association (EHA), and American Society of Clinical Oncology (ASCO) annual meetings, reference lists of included studies, and major pharmaceutical companies’ clinical trial databases to identify missed studies. The review was registered prospectively (INPLASY202630023). During revision the eligibility criteria were amended so that trials meeting all clinical criteria but reporting no usable time-to-event hazard ratio, or evaluating a multistage strategy that cannot be mapped onto a single exact-regimen node, are retained as narrative-only evidence and contribute to no quantitative synthesis. This amendment was made after registration and is reported here as a deviation from the registered protocol. Before resubmission, all full-text reports excluded in the original screening round were re-assessed against the amended criteria by two reviewers independently, and targeted study-level surveillance was performed for trials published between 22 November 2025 and 18 July 2026. Two previously excluded reports (BENEFIT/IFM2020–05 and GEM-2017FIT) became eligible under the revised criteria, and one post-cutoff trial (KMM1910) was identified; a second record-level database search, deduplication, and screening chain was not performed (**Figure 1**).

### 2.2 Eligibility criteria

Eligible studies were randomised controlled trials enrolling adults (≥18 years) with newly diagnosed multiple myeloma who were transplant-ineligible or were not scheduled for immediate autologous stem cell transplantation according to the trial protocol, and that compared at least two target first-line regimens. The primary outcome was progression-free survival; secondary outcomes were overall survival and grade ≥3 adverse events, and progression-free survival in the high-risk cytogenetic subgroup was analysed as an exploratory outcome. Studies were excluded if they were non-randomised, enrolled only patients intended for immediate transplantation, evaluated relapsed or refractory multiple myeloma or non-target regimens, were duplicate reports, or were published in languages other than English or Chinese. Trials meeting the clinical eligibility criteria but reporting no usable time-to-event hazard ratio, or evaluating a multistage treatment strategy that could not be represented as a single exact-regimen node, were retained for narrative synthesis only and contributed to no quantitative analysis. CEPHEUS enrolled a transplant-ineligible or transplant-deferred population; its full intention-to-treat estimate was used as the primary input, and the final transplant-ineligible subgroup estimates presented at the 2026 European Hematology Association Congress were analysed as a sensitivity analysis (**Supplementary Figure 13**). COBRA was excluded because its overall progression-free survival benefit was confined to transplant-eligible patients, its transplant-ineligible subgroup estimate was incompletely reported, and its multistage treatment strategies could not be mapped to the exact-regimen nodes used in the network.

### 2.3 Study selection and data extraction

Study selection was independently conducted by two reviewers (CT and CCL). Duplicates were first removed, followed by title/abstract screening and full-text assessment against inclusion/exclusion criteria. Disagreements were resolved via discussion, with a third reviewer (HYW) adjudicating if consensus was not reached. Zotero software was used for reference management, and the process is shown in a PRISMA flow diagram. For data extraction, two reviewers independently used a standardised form to extract study, population, intervention, and outcome data. When hazard ratio (HR) and 95% confidence interval (CI) were not directly reported, they were estimated using methods described by Tierney et al. or Guyot et al. Detailed data are presented in **Tables 1** and **2**.

**Table 1:** Characteristics of randomized trials included in the updated systematic review.

Trial	NCT number	Phase	Design	Setting	Primary endpoint	Comparison	N Total	Median follow-up (months)	Evidence role	Analysis status	Key inclusion criteria
IFM2017-03	NCT03993912	III	Open-label, randomized, multicenter	France	PFS	D-R–limited dexamethasone vs Rd	295	46.3	Strict primary time-to-event NMA	Final	Age ≥65, NDMM, simplified frailty score ≥2, transplant-ineligible
CEPHEUS	NCT03652064	III	Open-label, randomized, multicenter	Global	MRD negativity	D-VRd vs VRd	395	58.7	Strict primary time-to-event NMA	Peer-reviewed ITT primary; 2026 TIE-only sensitivity	NDMM, transplant-ineligible or transplant-deferred, age <80
SWOG S0777	NCT00644228	III	Open-label, randomized, multicenter	USA cooperative group	PFS	VRd vs Rd	525 (202 age ≥65)	55	Strict primary time-to-event NMA	Post hoc age ≥65 subgroup	NDMM, no intent for immediate ASCT
MAIA	NCT02252172	III	Open-label, randomized, multicenter	Global	PFS	D-Rd vs Rd	737	64.5	Strict primary time-to-event NMA	Long-term final	NDMM, transplant-ineligible
ENDURANCE	NCT01863550	III	Open-label, randomized, multicenter	USA cooperative group	PFS	KRd vs VRd	1087	~70	Strict primary time-to-event NMA	Long-term update	NDMM, no intent for immediate ASCT; standard-risk-enriched
IMROZ	NCT03319667	III	Open-label, randomized, multicenter	Global	PFS	Isa-VRd vs VRd	446	59.7	Strict primary time-to-event NMA	Primary PFS; OS immature	NDMM, transplant-ineligible, age ≤80
ALCYONE	NCT02195479	III	Open-label, randomized, multicenter	Global	PFS	D-VMP vs VMP	706	86.7	Extended-network sensitivity	Extended sensitivity; final OS	NDMM, transplant-ineligible; melphalan backbone
Real-MM	NCT03829371	IV	Open-label, randomized, multicenter	Italy, multicenter	PFS	VMP vs Rd	231 (114/117)	28.94	Extended-network sensitivity	Formal peer-reviewed randomized phase IV report	Pragmatic elderly/transplant-ineligible NDMM
KMM1910	NCT04277845	II	Open-label, randomized, multicenter	Korea, 14 centers	3-year PFS rate	VRd vs Rd	49	53.3	Systematic review only; narrative	Final; HR unavailable	Age ≥70, NDMM, ECOG 0–2, ASCT-ineligible
BENEFIT / IFM2020-05	NCT04751877	III	Open-label, randomized, multicenter	France / IFM	MRD negativity at 18 months	Isa-VRd vs Isa-Rd	270 (135/135)	23.5	Systematic review only; narrative	PFS/OS immature; HR unavailable	Non-frail transplant-ineligible NDMM
GEM-2017FIT	NCT03742297	III	Open-label, randomized, multicenter	Spain, 57 hospitals	MRD negativity after induction	VMP9–Rd9 vs KRd vs D-KRd	462	33.15	Systematic review only; multistage-strategy evidence	PFS reported; OS pending; NMA estimand incompatible	Age 65–80, GAH score ≤42, fit transplant-ineligible NDMM

The updated systematic review includes 11 randomized trials: six in the strict primary time-to-event NMA, ALCYONE and Real-MM in extended-network sensitivity analyses, and three narrative-only eligible RCTs.

Real-MM (NCT03829371) is a randomized phase IV trial, not observational evidence.

KMM1910 and BENEFIT lacked usable time-to-event HRs; GEM-2017FIT estimated an incompatible multistage treatment strategy.

**Table 2:** Efficacy outcomes and quantitative-synthesis roles of included randomized trials.

Trial	Comparison	N (experimental/control)	Analysis	PFS HR (reported CI) or narrative result	OS HR (reported CI) or narrative result	Quantitative role
IFM2017-03	D-R–limited dexamethasone vs Rd	200/95	Final	0.51 (0.37–0.70)	0.52 (0.35–0.77)	Strict primary time-to-event NMA
CEPHEUS	D-VRd vs VRd	197/198	Peer-reviewed ITT primary; TIE-only sensitivity	0.57 (0.41–0.79)	0.85 (0.58–1.24)	Strict primary time-to-event NMA
SWOG S0777	VRd vs Rd	93/109	Post hoc age ≥65 subgroup	0.83 (0.60–1.16)	0.83 (0.55–1.23)	Strict primary time-to-event NMA
MAIA	D-Rd vs Rd	368/369	Long-term final	0.55 (0.45–0.67)	0.66 (0.53–0.83)	Strict primary time-to-event NMA
ENDURANCE	KRd vs VRd	545/542	Long-term update	0.89 (0.76–1.04)	0.92 (0.75–1.12)	Strict primary time-to-event NMA
ALCYONE	D-VMP vs VMP	350/356	Extended sensitivity; final OS	0.42 (0.34–0.51)	0.65 (0.53–0.80)	Extended-network sensitivity
Real-MM	VMP vs Rd	114/117	Formal randomized phase IV report	0.93 (0.63–1.38)	0.52 (0.29–0.93)	Extended-network sensitivity
IMROZ	Isa-VRd vs VRd	265/181	Primary PFS; OS immature	0.596 (0.406–0.876)*	0.776 (0.407–1.480)†	Strict primary time-to-event NMA
KMM1910	VRd vs Rd	26/23 randomized	Final; early termination for slow accrual	Median 43.5 vs 24.8 mo; 3-y PFS 53.8% vs 45.5%; HR not reported	5-y OS 62.9% vs 63.6%; HR not reported	Systematic review only; no reliable HR/CI
BENEFIT / IFM2020-05	Isa-VRd vs Isa-Rd	135/135	Primary report; survival immature	Early PFS reported; usable HR/CI unavailable	Immature; usable HR/CI unavailable	Systematic review only; time-to-event data immature
GEM-2017FIT	KRd and D-KRd vs VMP9–Rd9	KRd 154; D-KRd 154; VMP9–Rd9 154 (one D-KRd patient later found ineligible)	Ongoing multistage phase 3 strategy	KRd: HR 0.58 (95% CI 0.38–0.88); D-KRd: HR 0.57 (0.37–0.89)	Not yet reported; planned separate publication	Systematic review only; multistage estimand incompatible with exact-regimen NMA

*For IMROZ, the PFS estimate is reported with the prespecified multiplicity-adjusted 98.5% CI; † the OS estimate is reported with the prespecified multiplicity-adjusted 99.97% CI.

Real-MM is a randomized phase IV trial; frozen extended-network inputs are PFS HR 0.93 (0.63–1.38) and main ITT OS HR 0.52 (0.29–0.93).

GEM-2017FIT PFS HRs are shown narratively but were not inserted into the exact-regimen NMA.

CEPHEUS TIE-only results remain sensitivity analyses; the peer-reviewed ITT effect remains primary.

### 2.4 Risk of bias assessment

Risk of bias in included RCTs was assessed using the Cochrane Risk of Bias 2.0 (RoB 2) tool (20). Each domain was rated as “low risk”, “some concerns”, or “high risk”. Overall risk of bias was determined by the worst-performing domain. Assessment was independently performed by two reviewers, with disagreements resolved through discussion or third-party adjudication.

### 2.5 Statistical analysis

Model selection: The primary analysis used a common-effect model. This follows from the geometry of the network rather than from a preference: the primary network is a loop-free tree in which every comparison is informed by a single trial, so there is no within-comparison replication and the between-study standard deviation is not identified by the data. Random-effects models were therefore fitted only as a prior-sensitivity layer, using half-normal priors on the between-study standard deviation with scales of 0.20, 0.50 and 1.00 (**Supplementary Figure 14**) rather than the informative predictive distributions of Turner et al. (21). Treatments were ranked by the surface under the cumulative ranking curve (SUCRA) in both the primary and the high-risk subgroup analyses. SUCRA summarises the posterior rank distribution and is reported here as a descriptive ranking only: it carries no interval estimate and is not used to declare one regimen superior to another.

Grade ≥3 adverse events were analysed separately by endpoint using a Bayesian binomial-logit model, with odds ratios (OR) as the effect measure. Eleven endpoints were modelled separately where the trials reported them: neutropenia, anaemia, thrombocytopenia, overall infection, pneumonia, lung infection, peripheral neuropathy, peripheral sensory neuropathy, diarrhoea, cardiac failure, and thromboembolic events. As SWOG S0777 did not report specific adverse event data, the evidence graph was built separately for each endpoint; a component that was connected and contained three or more nodes was analysed by network meta-analysis, whereas an endpoint informed by a single randomised comparison is reported as a direct pairwise estimate. No comparison is made across the Rd and VRd backbones, and no ranking spanning all regimens is produced. Common-effect models were used throughout. For a small number of infection endpoints in MAIA and IMROZ the trial reported a percentage rather than an event count; the numerator was reconstructed from the reported percentage and the safety-analysis denominator. Because rounding leaves this reconstruction uncertain by approximately one event, each affected endpoint was re-analysed with the reconstructed numerator varied by ±1 in the experimental arm, in the control arm, and in both, and again with the reconstructed trial removed. The direction and the significance of every affected estimate were unchanged.

High-risk subgroup analysis: HR values for PFS in the high-risk cytogenetics subgroup [del(17p), t(4;14), and/or t(14;16)] were extracted from each trial to construct an independent NMA network. ENDURANCE was excluded because it prospectively excluded patients with high-risk cytogenetics and therefore provided no eligible high-risk subgroup estimate. A common-effect model was used, for the same structural reason as in the primary analysis. Because the SWOG S0777 high-risk subgroup was the only link between the Rd and VRd backbones and contributed 6 and 11 patients, a sensitivity analysis excluding that trial was also performed; the network then separates into two components, which were analysed separately with no ranking across components. Bayesian models were fitted in R version 4.4.2 using JAGS through the rjags package (version 4-17), with posterior summaries and convergence diagnostics computed in coda (version 0.19-4.1); network topology was independently audited using igraph (version 2.2.2), and figures were produced with ggplot2 (version 4.0.2). Four chains were run with 10,000 adaptation iterations, 20,000 burn-in iterations, and 50,000 retained iterations per chain without thinning; where the convergence gate was not met, sampling was extended once by a further 50,000 iterations. Convergence was assessed by trace inspection and the Gelman–Rubin statistic. Random seeds, package versions, and convergence diagnostics are held with the frozen analysis objects and are available from the corresponding author together with the analysis code.

## 3 Results

### 3.1 Study selection

Literature screening was conducted in accordance with the PRISMA 2020 statement (22). We searched PubMed, Embase, Cochrane Library, ClinicalTrials.gov, and relevant reference lists, identifying 3003 records (2847 from databases, 156 from other sources). After Zotero-assisted deduplication, 2134 records were screened by title and abstract, with 2012 excluded (mainly non-RCTs, wrong population/intervention). The remaining 122 full texts were reviewed. After all excluded full-text reports were re-assessed against the revised eligibility criteria, 112 were excluded and 10 randomised trials were included from the original full-text set; targeted study-level surveillance identified one further eligible trial published after the search cutoff (KMM1910), giving 11 in total. Of these, 6 (IFM2017-03 (23), CEPHEUS (14), SWOG S0777 ≥65 years subgroup (24), MAIA (12, 13), ENDURANCE (16), IMROZ (15)) were included in the NMA. The Real-MM trial (25), a randomised phase IV trial enrolling a broader and more comorbid population, entered the extended-network sensitivity analysis by providing the VMP–Rd link. Because this was the only connection between the VMP and Rd components, the extended-network findings were considered exploratory. ALCYONE (26, 27), which compares D-VMP with VMP and is not connected to the primary network, entered the extended-network sensitivity analysis through the VMP–Rd comparison provided by Real-MM. The COBRA trial (NCT03729804, KRd versus VRd) was assessed at full text but excluded because: (1) it enrolled a mixed transplant-eligible and transplant-ineligible population, and the progression-free survival benefit in the overall population was confined to transplant-eligible patients (transplant-ineligible subgroup: HR = 1.06, P = 0.87); (2) the two arms represented asymmetric multistage treatment strategies—KRd for 24 cycles versus VRd for 8 cycles followed by Rd maintenance—that could not be mapped to the same exact-regimen nodes used in the primary network; and (3) only abstract-level interim results were available, with immature overall survival data and incomplete reporting of the transplant-ineligible subgroup, including the absence of a confidence interval for its progression-free survival estimate. The selection process is shown in **Figure 1**. The three trials contributing only to narrative synthesis—KMM1910 (VRd vs Rd) (28), BENEFIT/IFM2020-05 (Isa-VRd vs Isa-Rd) (29), and GEM-2017FIT (VMP9–Rd9 vs KRd vs D-KRd) (30)—are summarised narratively in **Table 1** and contribute to no quantitative synthesis: the first two report no usable time-to-event hazard ratio, and the third evaluates a multistage treatment strategy that cannot be mapped onto a single exact-regimen node.

### 3.2 Study characteristics and quality assessment

**Figure 2:**
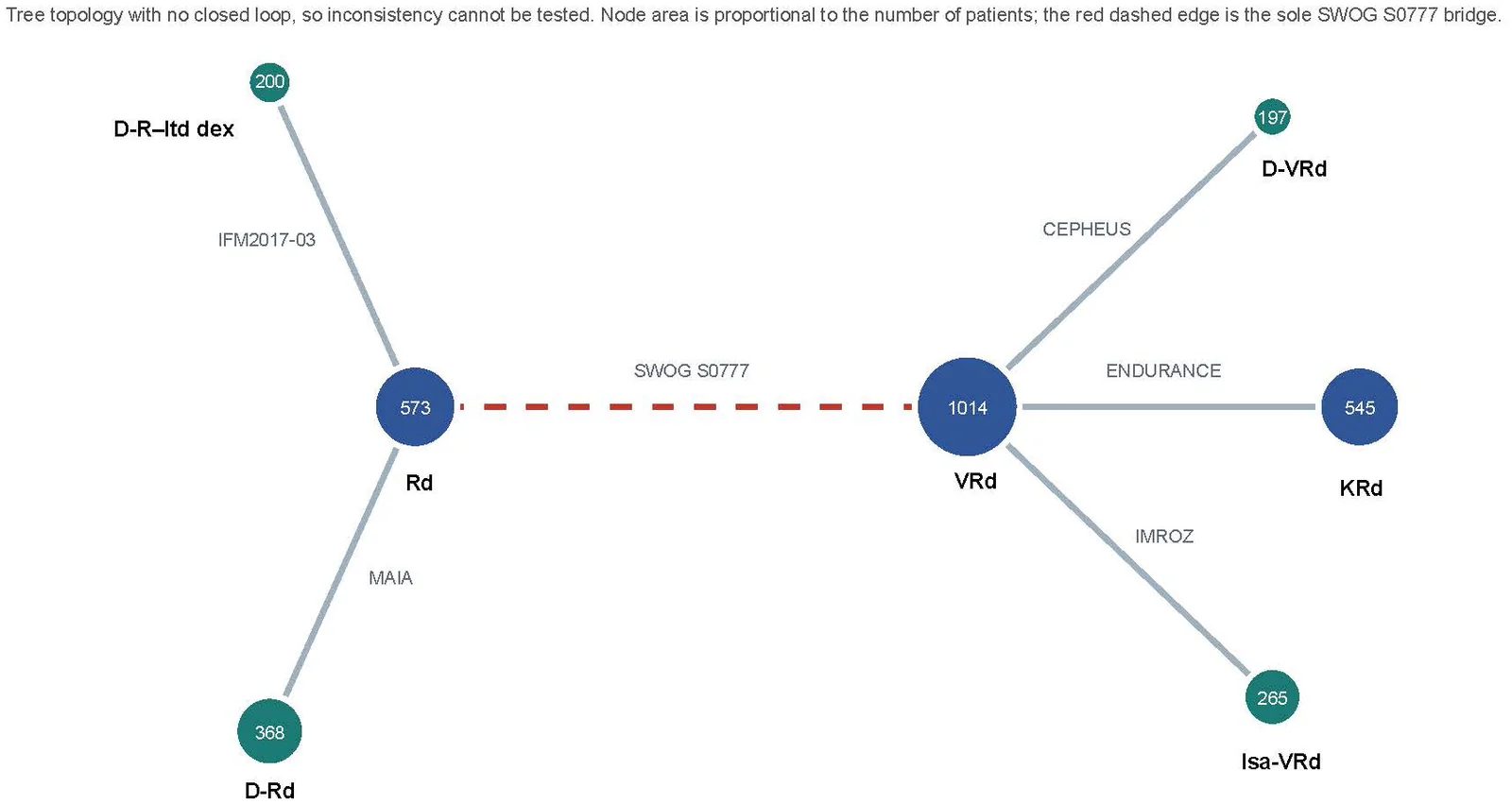
Network of treatment comparisons. Node size proportional to sample size; edge width proportional to total participants. *SWOG S0777: age ≥65 years subgroup.

The primary analysis included 6 RCTs encompassing 7 treatment nodes and 3162 patients. The treatment network formed a loop-free, two-hub tree with Rd and VRd as the central nodes: D-R–limited dexamethasone (n=200) from IFM2017–03 and D-Rd (n=368) from MAIA connected through Rd; D-VRd (n=197), Isa-VRd (n=265), and KRd (n=545) connected through VRd; and SWOG S0777 providing bridging evidence between Rd and VRd (**Figure 2**). The VRd node had the largest sample size (n=1014), reflecting its role as the control arm in multiple trials.

The 6 trials contributing to the primary network, together with the other eligible randomised trials, were assessed using the RoB 2.0 tool across 5 domains (**Supplementary Figure 1**). All trials had an open-label design, which contributed to “some concerns” in one or more domains in several of the trials, although CEPHEUS, MAIA and IMROZ were rated low risk in every domain. CEPHEUS, MAIA and IMROZ were rated low risk overall; IFM2017–03 and ENDURANCE raised some concerns; and SWOG S0777 was rated high risk overall, driven by the domain for selection of the reported result. Because SWOG S0777 is the sole bridge between the Rd and VRd backbones, its high risk of bias reduces confidence in every cross-backbone indirect comparison. Because none of the trials was blinded, risk of bias in outcome measurement was judged by how the outcome was ascertained: progression-free survival was determined by an independent review committee or a validated computerised algorithm in MAIA, IMROZ, CEPHEUS and ALCYONE (rated low risk in that domain), and by investigator assessment in IFM2017-03, ENDURANCE, SWOG S0777 and Real-MM (rated some concerns). Adverse events were reported by unblinded investigators in every trial and are additionally sensitive to differences in exposure duration, ascertainment intensity and reporting thresholds; the safety estimates are therefore used only to identify regimen-specific signals within each trial’s own randomised comparison and are not treated as confirmatory. The six direct trial-level PFS estimates are displayed in a funnel plot for completeness (**Supplementary Figure 3**). Because each trial contributes a different randomised comparison and the network contains no closed loop, this display cannot be interpreted as a comparison-adjusted funnel plot; no pooled effect is shown and no formal small-study-effects test was performed.

### 3.3 Patient baseline characteristics

Baseline characteristics of the 6 trials contributing to the primary network are summarised in **Table 3**, with well-balanced treatment groups. Each arm included 95 to 545 patients with reasonable allocation ratios; the SWOG S0777 age ≥65 subgroup that entered the efficacy analysis contributed 93 and 109 patients and is described in the **Table 3** footnote. Median age ranged from 63 to 81 years. A total of 56.5%–92.9% of patients had an ECOG performance status of 0–1, with the lowest proportion in IFM2017–03 and the highest in CEPHEUS, reflecting different fitness profiles across trials. Most patients had ISS stage I–II disease (65.5%–88.3%) and standard-risk cytogenetics (58.9%–100%). SWOG S0777 used the ≥65-year subgroup for efficacy analysis; as subgroup-specific baseline characteristics were not reported, total population baseline data from SWOG S0777 (31) were used as an approximation. Treatment arms were generally balanced within each trial; clinically relevant differences between trials in age, frailty, transplant-eligibility criteria, and follow-up maturity nevertheless remained and were considered in the transitivity assessment.

**Table 3:** Baseline characteristics of randomized trials contributing to the primary time-to-event NMA.

Characteristic	IFM2017-03		CEPHEUS		SWOG S0777*		MAIA		ENDURANCE		IMROZ	
Characteristic	D-R–limited dexamethasone	Rd	D-VRd	VRd	VRd	Rd	D-Rd	Rd	KRd	VRd	Isa-VRd	VRd
No. of patients	200	95	197	198	242	229	368	369	545	542	265	181
Age, median (IQR or range), yr	81 (77–84)‡	81 (77–84)‡	70 (42–79)	70 (31–80)	63 (56–70)‡	63 (56–71)‡	73 (50–90)	74 (45–89)	65 (59–71)	64 (57–71)	72 (60–80)	72 (55–80)
Female sex — no. (%)	102 (51.0)	49 (51.6)	110 (55.8)	87 (43.9)	89 (36.8)	107 (46.7)	179 (48.6)	174 (47.2)	218 (40.0)	227 (41.9)	122 (46.0)	87 (48.1)
ECOG PS 0–1 — no. (%)	113 (56.5)	57 (60.0)	174 (88.3)	184 (92.9)	214 (88.4)	193 (84.3)	305 (82.9)	310 (84.0)	490 (89.9)	482 (88.9)	235 (88.7)	162 (89.5)
ECOG PS ≥2 — no. (%)	87 (43.5)	38 (40.0)	23 (11.7)	14 (7.1)	28 (11.6)	36 (15.7)	63 (17.1)	59 (16.0)	55 (10.1)	60 (11.1)	30 (11.3)	19 (10.5)
ISS stage — no. (%)												
Stage I or II	136 (68.0)	68 (71.6)	141 (71.6)	143 (72.2)	164 (67.8)	150 (65.5)	261 (70.9)	259 (70.2)	383 (70.3)	401 (74.0)	234 (88.3)	157 (86.7)
Stage III	64 (32.0)	27 (28.4)	56 (28.4)	55 (27.8)	78 (32.2)	79 (34.5)	107 (29.1)	110 (29.8)	159 (29.3)	138 (25.5)	29 (10.9)	21 (11.6)
Cytogenetic risk — no./total no. (%)												
Standard risk	146 (73.0)	56 (58.9)	149 (75.6)	149 (75.3)	NR	NR	271/319 (85.0)	279/323 (86.4)	545 (100)	542 (100)	207 (78.1)	140 (77.3)
High risk§	28 (14.0)	17 (17.9)	25 (12.7)	27 (13.6)	NR	NR	48/319 (15.0)	44/323 (13.6)	Excluded by design	Excluded by design	40 (15.1)	34 (18.8)

*SWOG S0777 efficacy estimates used the post hoc age ≥65 subgroup (93 VRd/109 Rd); baseline characteristics shown here are from the total-trial evaluable baseline population (242 VRd/229 Rd). Arm-specific cytogenetic-risk counts were not reported; in the overall evaluable cohort, 212/316 (67.1%) had standard-risk and 104/316 (32.9%) had high-risk cytogenetics.

‡Interquartile range reported instead of full range.

§High-risk cytogenetics were defined as del(17p), t(4;14), and/or t(14;16).

The IFM2017-03 high-risk count was 28 at baseline; the PFS subgroup included 31 patients with evaluable cytogenetics.

Scope: ALCYONE, Real-MM, KMM1910, BENEFIT, and GEM-2017FIT are summarized in the supplementary eligibility-and-synthesis table.

D-R–limited dexamethasone, daratumumab plus lenalidomide with dexamethasone limited to cycles 1–2; D-Rd, daratumumab plus lenalidomide and dexamethasone; D-VRd, daratumumab plus bortezomib, lenalidomide, and dexamethasone; Isa-VRd, isatuximab plus VRd; KRd, carfilzomib plus lenalidomide and dexamethasone; Rd, lenalidomide plus dexamethasone; VRd, bortezomib plus lenalidomide and dexamethasone.

### 3.4 Efficacy

Efficacy was systematically assessed through both direct comparisons and NMA across 6 RCTs and 7 treatment regimens (D-R–limited dexamethasone, D-Rd, D-VRd, Isa-VRd, VRd, KRd, Rd) for PFS and OS.

**Figure 3:**
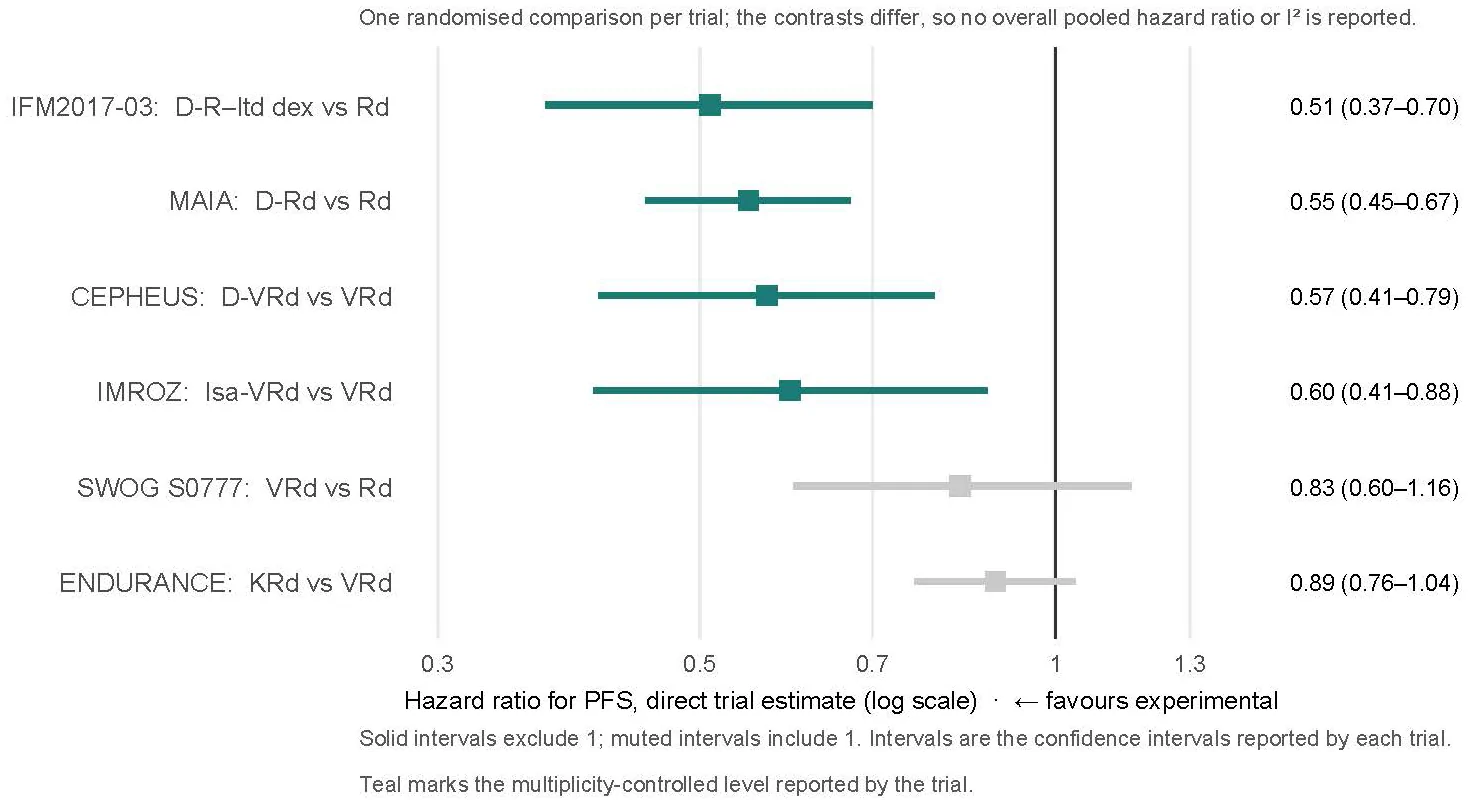
Forest plot: progression-free survival. Each trial contributes a different randomised comparison; no overall pooled HR is reported. ENDURANCE: KRd vs VRd; SWOG S0777: VRd vs Rd; IMROZ: Isa-VRd vs VRd; CEPHEUS: D-VRd vs VRd; MAIA: D-Rd vs Rd; IFM2017-03: D-R–limited dexamethasone vs Rd.

PFS direct comparisons: Each trial contributed a different randomised comparison, so no overall pooled HR or heterogeneity statistic is reported (**Figure 3**). IFM2017–03 showed the most pronounced PFS benefit (HR = 0.51, 95% CI: 0.37–0.70), followed by MAIA (HR = 0.55, 95% CI: 0.45–0.67), while ENDURANCE showed no statistically significant difference (HR = 0.89, 95% CI: 0.76–1.04).

**Figure 4:**
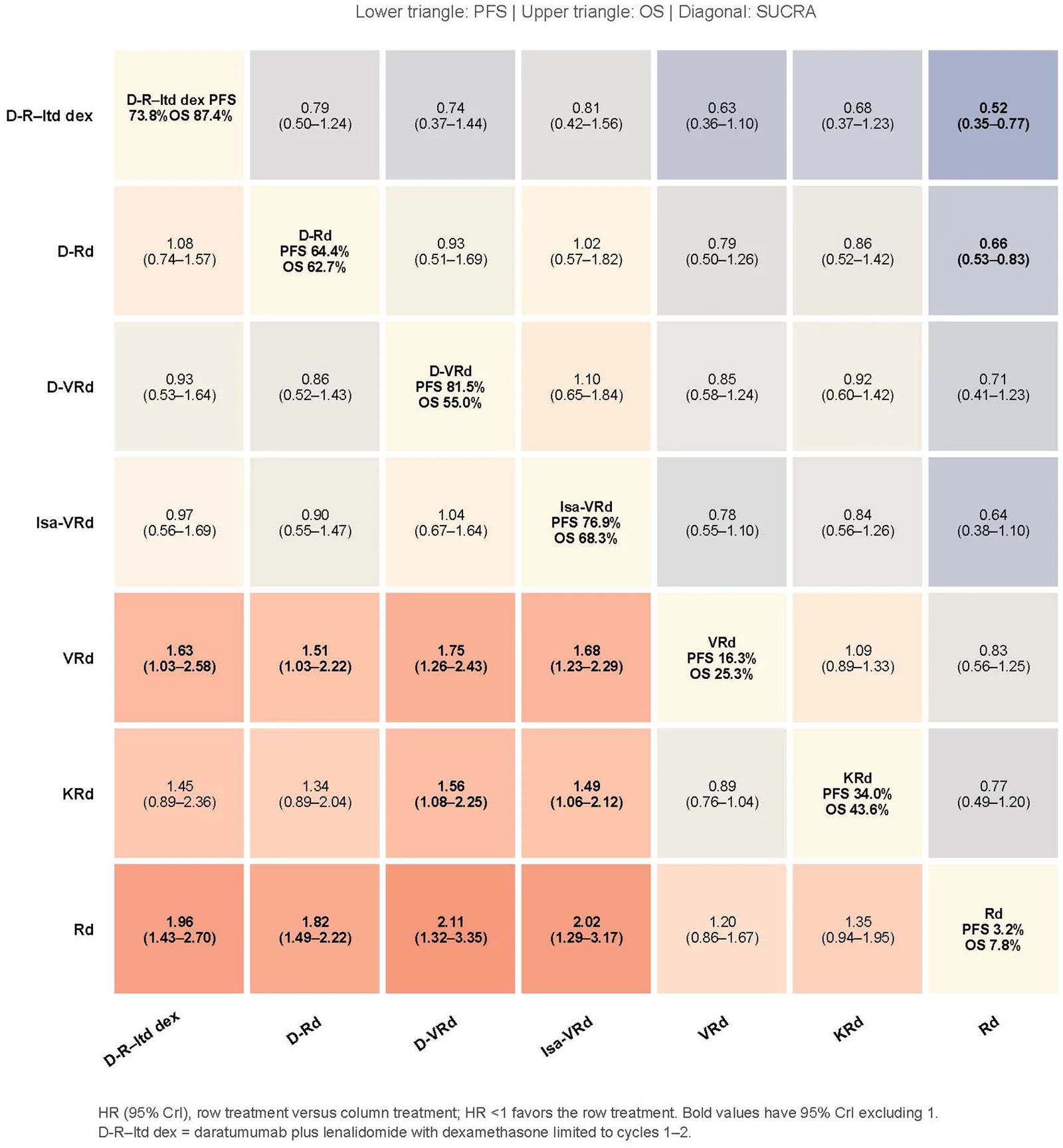
League table: network meta-analysis results. Lower triangle: PFS; Upper triangle: OS; Diagonal: SUCRA. HR (95% CrI); row versus column; HR<1 favours the row treatment. Bold values have a 95% CrI excluding 1.

PFS network meta-analysis: All four anti-CD38-containing regimens showed favourable PFS estimates relative to Rd (**Figure 4**; lower triangle; **Supplementary Figure 6A**). Compared with Rd, D-R–limited dexamethasone (HR = 0.51, 95% CrI: 0.37–0.70), D-Rd (HR = 0.55, 95% CrI: 0.45–0.67), D-VRd (HR = 0.47, 95% CrI: 0.30–0.76), and Isa-VRd (HR = 0.49, 95% CrI: 0.32–0.78) each reduced the risk of progression or death by approximately 45–53%. The corresponding estimates versus VRd were 0.61 (95% CrI: 0.39–0.97), 0.66 (0.45–0.97), 0.57 (0.41–0.79), and 0.60 (0.44–0.81). KRd did not differ significantly from VRd (HR = 0.89, 95% CrI: 0.76–1.04). No comparison among the four anti-CD38-containing regimens reached significance; for example, Isa-VRd versus D-VRd was 1.04 (95% CrI: 0.67–1.64) and D-Rd versus D-R–limited dexamethasone was 1.08 (95% CrI: 0.74–1.57). Because the network is a loop-free tree, the point estimate for any directly compared pair reproduces that trial’s own estimate. The credible interval need not coincide with the published interval: IMROZ reported its primary progression-free survival result with a multiplicity-adjusted 98.5% confidence interval, which was converted to a standard error at that level before entering the model.

**Figure 5:**
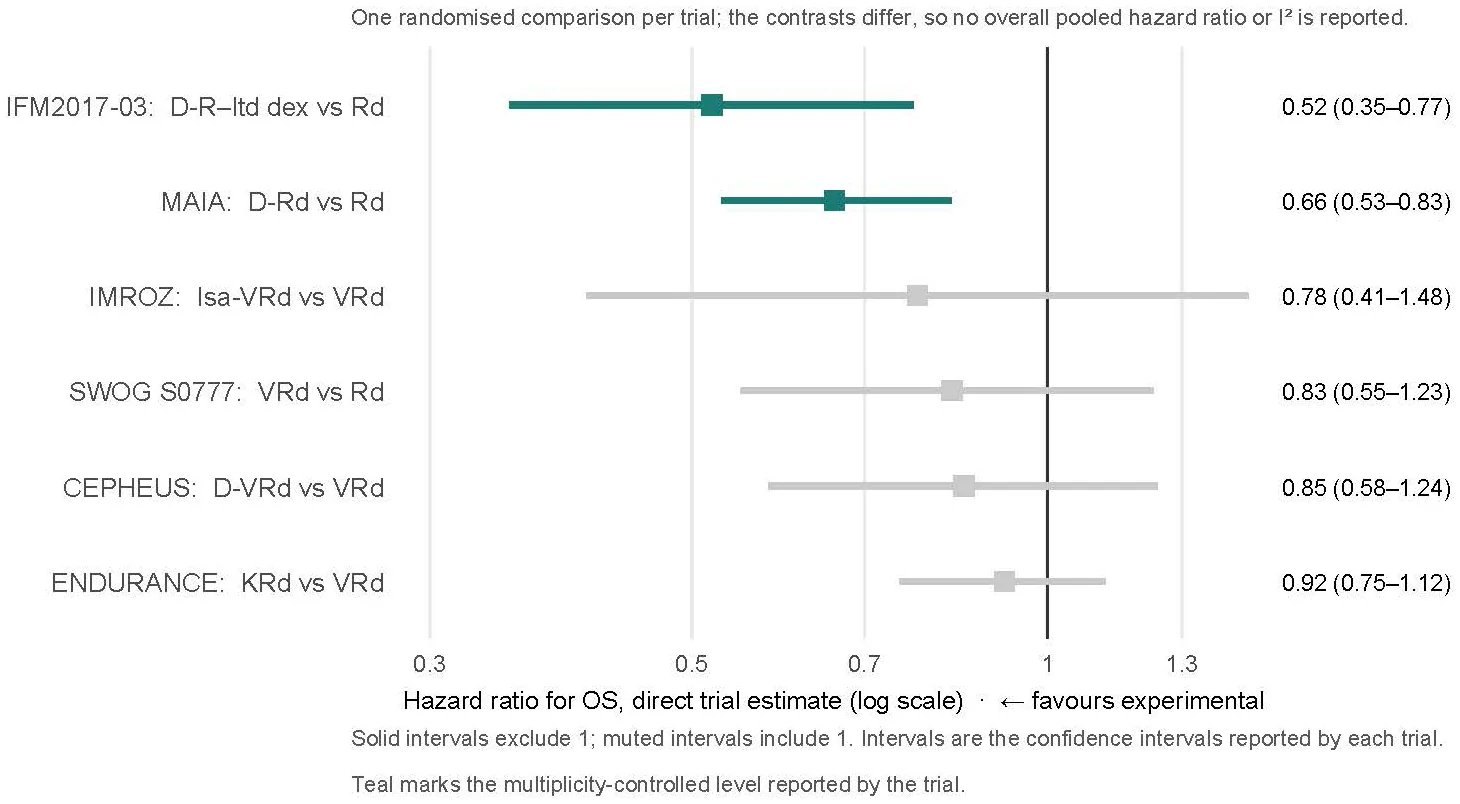
Forest plot: overall survival. Each trial contributes a different randomised comparison; no overall pooled HR is reported.

OS direct comparisons: Each trial contributed a different randomised comparison, so no overall pooled HR or heterogeneity statistic is reported (**Figure 5**). IFM2017–03 showed the most significant OS benefit (HR = 0.52, 95% CI: 0.35–0.77), MAIA also reached statistical significance (HR = 0.66, 95% CI: 0.53–0.83), while the remaining trials did not achieve statistical significance.

OS network meta-analysis: NMA results (**Figure 4**; upper triangle; **Supplementary Figure 6B**) showed that only the two daratumumab–lenalidomide regimens reached significance versus Rd: D-R–limited dexamethasone (HR = 0.52, 95% CrI: 0.35–0.77) and D-Rd (HR = 0.66, 95% CrI: 0.53–0.83). Neither quadruplet did (D-VRd vs Rd: HR = 0.71, 95% CrI: 0.41–1.23; Isa-VRd vs Rd: HR = 0.64, 95% CrI: 0.38–1.10), nor did VRd (HR = 0.83, 95% CrI: 0.56–1.25) or KRd (HR = 0.77, 95% CrI: 0.49–1.20). No comparison among the anti-CD38-containing regimens reached significance. OS follow-up is considerably less mature for the two quadruplet trials than for MAIA and IFM2017-03, so these contrasts should not be read as evidence of equivalence.

Treatment ranking: SUCRA analysis (**Supplementary Figure 2**) showed the following PFS ranking: D-VRd (81.5%), Isa-VRd (76.9%), D-R–limited dexamethasone (73.8%), D-Rd (64.4%), KRd (34.0%), VRd (16.3%), and Rd (3.2%). The OS ranking was: D-R–limited dexamethasone (87.4%), Isa-VRd (68.3%), D-Rd (62.7%), D-VRd (55.0%), KRd (43.6%), VRd (25.3%), and Rd (7.8%). D-VRd ranked first for PFS whereas D-R–limited dexamethasone ranked first for OS. SUCRA is a point summary of the posterior rank distribution and carries no measure of uncertainty; the rankogram (**Supplementary Figure 5**) shows that the rank distributions of the four anti-CD38-containing regimens overlap extensively, so this ordering should not be read as a demonstrated difference between them.

### 3.5 Safety analysis

Distinct safety profiles were observed across regimens. The safety heatmap (**Supplementary Figure 7**) reports each randomised arm of each trial separately, with the event count and denominator shown; percentages are deliberately not pooled across trials, because follow-up duration, ascertainment, and reporting thresholds differ. Grade ≥3 neutropenia was the most common high-grade toxicity in every anti-CD38-containing arm (55% with D-R–limited dexamethasone in IFM2017-03, 54% with D-Rd in MAIA, 54% with Isa-VRd in IMROZ, and 44% with D-VRd in CEPHEUS), against 24–37% in the corresponding control arms. Thrombocytopenia was highest in the quadruplet arms and peripheral sensory neuropathy lowest with KRd, reflecting the neurotoxicity advantage of carfilzomib over bortezomib. The largest arm-level proportions for overall infection were seen with Isa-VRd (45%) in IMROZ and D-Rd (43%) in MAIA; arm-level proportions are not directly comparable across trials, and in the modelled estimates only D-Rd increased overall infection relative to its own control.

Odds ratios with 95% credible intervals (CrI) for every endpoint are shown in **Supplementary Figure 12**. Because SWOG S0777 reported no endpoint-specific adverse event data, the safety evidence separates into an Rd-backbone component, a VRd-backbone component, and several contrasts informed by a single trial; no comparison is made across components. Within the Rd backbone the two daratumumab–lenalidomide regimens behaved differently: D-R–limited dexamethasone increased neutropenia (OR = 3.85, 95% CrI: 2.26–6.74) and anaemia (OR = 4.24, 95% CrI: 1.45–17.70) but not pneumonia (OR = 0.61, 95% CrI: 0.25–1.57) or overall infection (OR = 0.88, 95% CrI: 0.48–1.64), whereas D-Rd increased neutropenia (OR = 2.01, 95% CrI: 1.49–2.71), pneumonia (OR = 2.03, 95% CrI: 1.34–3.09), overall infection (OR = 1.77, 95% CrI: 1.30–2.39), and peripheral sensory neuropathy (OR = 4.51, 95% CrI: 1.16–27.97). Within the VRd backbone, D-VRd (OR = 1.87, 95% CrI: 1.24–2.84) and Isa-VRd (OR = 2.03, 95% CrI: 1.38–2.99) both increased neutropenia, and Isa-VRd also increased pneumonia (OR = 1.74, 95% CrI: 1.03–3.01); neither increased overall infection. KRd markedly reduced peripheral sensory neuropathy (OR = 0.07, 95% CrI: 0.02–0.19) but, on single-trial evidence from ENDURANCE, increased cardiac failure (OR = 3.27, 95% CrI: 1.36–8.93), lung infection (OR = 3.13, 95% CrI: 1.50–7.06), and thromboembolic events (OR = 2.44, 95% CrI: 1.22–5.18). The widest intervals correspond to the sparsest event counts and should not be read as precise risk estimates.

### 3.6 Consistency assessment

The primary network is a loop-free tree in which every comparison is informed by a single trial; no direct-versus-indirect contrast is estimable, so node-splitting and formal inconsistency testing are not applicable (**Supplementary Figure 4**). The consistency assumption was therefore addressed through the transitivity assessment rather than statistically. Sensitivity analysis including ALCYONE and Real-MM extended the network to 9 treatment nodes (**Supplementary Figure 8**); the extended network also remained acyclic, so node-splitting was again not estimable. Alternative SWOG S0777 estimands—covariate-adjusted, and reconstructed from the published survival curves rather than from individual patient data—left the four anti-CD38-containing regimens as the top four and all of them above KRd, VRd and Rd; the internal order of those four did move, with D-R–limited dexamethasone ranking first under the covariate-adjusted estimand and second under the reconstructed-data estimand, compared with third in the primary analysis (**Supplementary Figure 10**). That instability is a further reason not to read the ranking as a demonstrated ordering. Replacing the CEPHEUS randomised intention-to-treat estimate with the final transplant-ineligible subgroup estimate left the progression-free survival ranking unchanged (D-VRd remained first) and left the all-cause overall survival ranking unchanged (D-R–limited dexamethasone remained first). In an exploratory analysis censoring COVID-19 deaths, D-VRd moved from fourth to second for overall survival; that analysis changes the estimand and does not substitute for all-cause overall survival (**Supplementary Figure 13**). Fitting random-effects models with half-normal priors of increasing scale left the point estimates essentially unchanged and progressively widened the credible intervals, as expected when the between-study standard deviation is informed only by its prior (**Supplementary Figure 14**).

### 3.7 High-risk cytogenetics subgroup analysis

The high-risk cytogenetics subgroup NMA included 5 trials (IFM2017-03, MAIA, SWOG S0777, CEPHEUS, IMROZ); ENDURANCE was excluded owing to its systematic exclusion of high-risk patients. High-risk was defined as del(17p), t(4;14), and/or t(14;16). Subgroup sample sizes were small, ranging from 6 to 48 patients per arm (**Supplementary Figure 9**). Results (**Supplementary Figure 11**) ranked D-R–limited dexamethasone highest (SUCRA 87%), followed by D-Rd (70%), D-VRd (45%), Isa-VRd (37%), VRd (32%), and Rd (29%). In the network, both daratumumab–lenalidomide regimens reduced progression risk relative to Rd (D-R–limited dexamethasone HR 0.42, 95% CrI 0.20–0.87; D-Rd HR 0.59, 95% CrI 0.44–0.80), whereas the quadruplet estimates were compatible with benefit, no effect, or harm (D-VRd versus VRd HR 0.88, 95% CrI 0.42–1.85; Isa-VRd versus VRd HR 0.97, 95% CrI 0.48–1.96). Because the network is a loop-free tree, the two Rd-backbone contrasts are identified by their own trials and do not use the SWOG S0777 bridge; excluding that trial split the network into two components and left them unchanged to within Monte Carlo error (0.42, 95% CrI 0.20–0.87 and 0.59, 95% CrI 0.44–0.79). Cross-backbone estimates were far less informative because the only bridge between backbones is the SWOG S0777 high-risk subgroup, which contributes 6 and 11 patients, and no ranking across the two components is estimable once it is removed. Given the extremely small subgroup sample sizes, lack of statistically significant differences among regimens, and the fact that the direct VRd versus Rd evidence came from a single trial (SWOG S0777; HR = 1.02, 95% CI: 0.30–3.20, with very wide confidence intervals) limiting the precision of indirect comparisons, these results should be considered exploratory.

## 4 Discussion

This NMA compared seven exact-regimen nodes across six randomised trials in transplant-ineligible NDMM. Its principal contribution is not to re-establish the class benefit of anti-CD38 therapy, but to quantify the relative evidence for specific regimens, preserve the dexamethasone-sparing IFM2017–03 strategy as a distinct node, distinguish PFS ranking from OS evidence maturity, and define the limitations of the available safety and high-risk subgroup evidence.

Because multiple anti-CD38-containing regimens are already recommended as first-line options (10, 32, 33), the clinically relevant question is whether the available evidence can distinguish among specific treatment strategies. All four anti-CD38-containing regimens improved PFS relative to Rd, but no pairwise comparison among them reached statistical significance and their posterior rank distributions overlapped substantially. The network therefore supports these regimens as effective first-line options but does not establish a preferred regimen. The common-effect model was used because every network edge was informed by a single trial; random-effects models were retained only to examine sensitivity to alternative heterogeneity priors.

The difference between the PFS and OS rankings should be interpreted mainly in light of evidence maturity. MAIA (13) and IFM2017-03 (23) had longer follow-up and more mature OS data than CEPHEUS (14) and IMROZ (15). Although PFS has been evaluated as a surrogate endpoint in multiple myeloma (34, 35), the present PFS ranking should not be assumed to predict eventual OS ordering. No pairwise OS comparison among the anti-CD38-containing regimens reached statistical significance; longer follow-up is therefore required before the PFS rankings of the quadruplet regimens can be translated into claims of long-term survival advantage.

Safety findings were endpoint- and regimen-specific. Anti-CD38-containing regimens consistently increased grade ≥3 neutropenia, whereas infection signals were not uniform across regimens. Bortezomib-containing regimens showed more peripheral neuropathy than KRd, whereas KRd showed higher cardiac-failure, lung-infection, and thromboembolic signals in ENDURANCE. Because adverse-event definitions, exposure duration, follow-up, and reporting differed across trials—and SWOG S0777 provided no compatible endpoint-specific safety data—these findings should guide targeted monitoring rather than be interpreted as a complete cross-regimen safety ranking. Infection prevention and toxicity monitoring should follow established myeloma guidance (36).

The high-risk analysis separates into a part that is interpretable and a part that is not. Because the network is a loop-free tree, the two daratumumab–lenalidomide contrasts against Rd are identified by their own trials and do not use the SWOG S0777 bridge: D-R–limited dexamethasone HR 0.42 (95% CrI 0.20–0.87) and D-Rd HR 0.59 (95% CrI 0.44–0.80), unchanged when that trial is removed. Two independent trials therefore point the same way in high-risk patients, including under a dexamethasone-sparing schedule in a frail population. These nevertheless remain trial-level subgroup analyses, which are generally neither powered nor multiplicity-controlled for the subgroup. What the analysis cannot do is rank regimens against one another: the quadruplet estimates were imprecise (D-VRd versus VRd HR 0.88, 95% CrI 0.42–1.85; Isa-VRd versus VRd HR 0.97, 95% CrI 0.48–1.96), every cross-backbone comparison depends on the SWOG S0777 bridge of 6 versus 11 patients, and D-R–limited dexamethasone versus D-Rd was indeterminate (HR 0.71, 95% CrI 0.32–1.57). Whether anti-CD38 therapy overcomes the adverse prognosis of high-risk cytogenetics therefore remains undetermined and would require trials powered for this subgroup.

Treatment selection should be guided primarily by fitness, frailty, comorbidities, toxicity susceptibility, treatment burden, and the populations enrolled in the direct randomised trials rather than by SUCRA ranking alone. Quadruplet regimens may be appropriate for selected fit patients, whereas the daratumumab–lenalidomide trials currently provide the longest OS follow-up and IFM2017-03 (23) supports a dexamethasone-sparing option for frail patients. The present indirect comparisons do not establish a universally preferred regimen.

Several limitations constrain interpretation. The primary network was a loop-free tree, so consistency could not be assessed statistically and most regimen comparisons relied entirely on indirect evidence. All trials were open label, and differences in eligibility, frailty, dexamethasone exposure, follow-up maturity, and outcome ascertainment may challenge transitivity. Each direct comparison was informed by a single trial, precluding estimation of within-comparison heterogeneity. OS was immature in some trials; safety analyses used inconsistently reported cumulative incidences without adjustment for exposure time; high-risk subgroups were small and variably defined; and aggregate data limited patient-level effect-modifier analyses. Generalisability to Asian populations also remains uncertain. The extended network was fragile because Real-MM provided the only VMP–Rd link (25). Safety findings are presented as regimen- and endpoint-specific signals rather than as comparable absolute risks, and no regimen ranking on safety is produced.

Souto Filho et al. reported a reconstructed individual-patient-data NMA of four randomised trials, finding an OS advantage for quadruplet versus triplet regimens (37). Unlike that analysis, our network retained IFM2017–03 as a distinct dexamethasone-sparing node, evaluated safety endpoint by endpoint, and included a high-risk subgroup analysis. However, differences between the two studies reflect both the included evidence and the assumptions required to reconstruct individual-level data from published survival curves; they should not be interpreted as new head-to-head evidence.

## 5 Conclusions

For transplant-ineligible NDMM patients, anti-CD38-based regimens improved PFS relative to Rd and are appropriate first-line therapy. They showed advantages for different outcomes: D-VRd ranked first for PFS (SUCRA 81.5%) and D-R–limited dexamethasone first for OS (SUCRA 87.4%), but no pairwise comparison among them reached significance. Safety analysis indicated that several anti-CD38-containing regimens increase grade ≥3 neutropenia, whereas infection signals were regimen- and endpoint-specific, necessitating enhanced monitoring and prophylactic management in clinical practice. The high-risk analysis could not rank regimens against one another, but the two daratumumab–lenalidomide regimens retained a progression-free survival benefit versus Rd within their own randomised comparisons; whether any regimen overcomes the adverse prognosis of high-risk cytogenetics requires trials powered for this subgroup. Preserving the dexamethasone-sparing schedule as a distinct node, and reporting adverse events endpoint by endpoint, may be useful for future analyses in this setting.

## Data Availability

The original contributions presented in the study are included in the article/**Supplementary Material**. Further inquiries can be directed to the corresponding authors.
