# Supplementary material for: First-Line treatment for transplant-ineligible newly diagnosed multiple myeloma: a Bayesian network meta-analysis of efficacy, safety, and high-risk subgroups
Source: Front Immunol. 2026 Aug 24;17:1869797. doi: 10.3389/fimmu.2026.1869797 (PMC13547454; doi:10.3389/fimmu.2026.1869797)
Supplement: Supplementary file 1 [file DataSheet1.pdf]

eFigure 1. Risk-of-bias assessment (RoB 2) for progression-free survival

Two-reviewer adjudicated consensus; effect of assignment to intervention (intention-to-treat estimand)

Study	D1	D2	D3	D4	D5	Overall
IFM2017-03	S	L	L	S	L	S
CEPHEUS	L	L	L	L	L	L
SWOG S0777	L	L	L	S	H	H
MAIA	L	L	L	L	L	L
ENDURANCE	L	L	L	S	L	S
IMROZ	L	L	L	L	L	L
ALCYONE	L	L	L	L	L	L
Real-MM	S	L	L	S	L	S
KMM1910	L	S	L	S	S	S
BENEFIT / IFM2020-05	L	L	L	S	S	S
GEM-2017FIT	S	L	S	L	L	S

RoB 2 domains

D1: Bias arising from the randomization process
D2: Bias due to deviations from intended interventions
D3: Bias due to missing outcome data
D4: Bias in measurement of the outcome
D5: Bias in selection of the reported result

Judgment legend

L Low risk
S Some concerns
H High risk

Overall judgments reflect the final adjudicated consensus of two independent reviewers.

eFigure 2. Treatment rankings by SUCRA (common-effect Bayesian NMA)

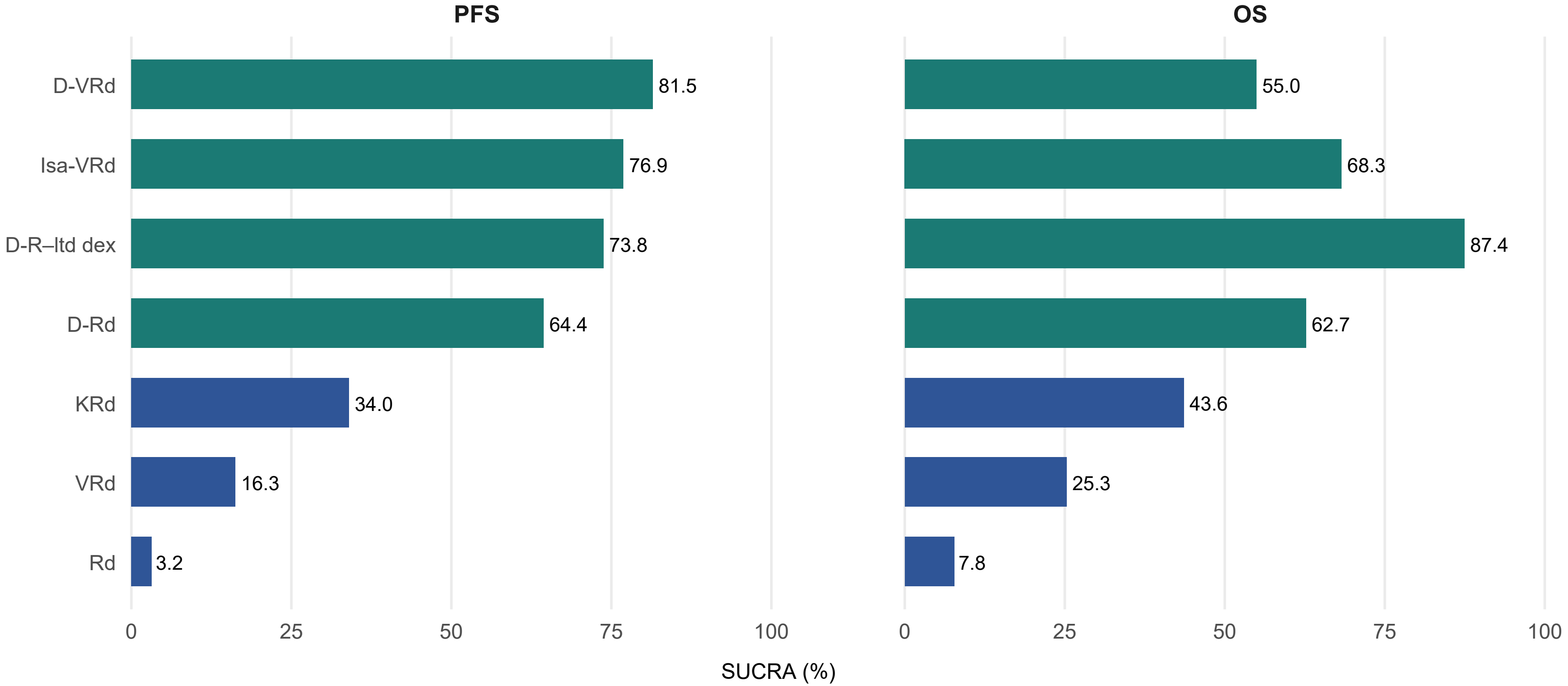

eFigure 3. Funnel plot of direct trial-level PFS estimates (primary 6-RCT network)

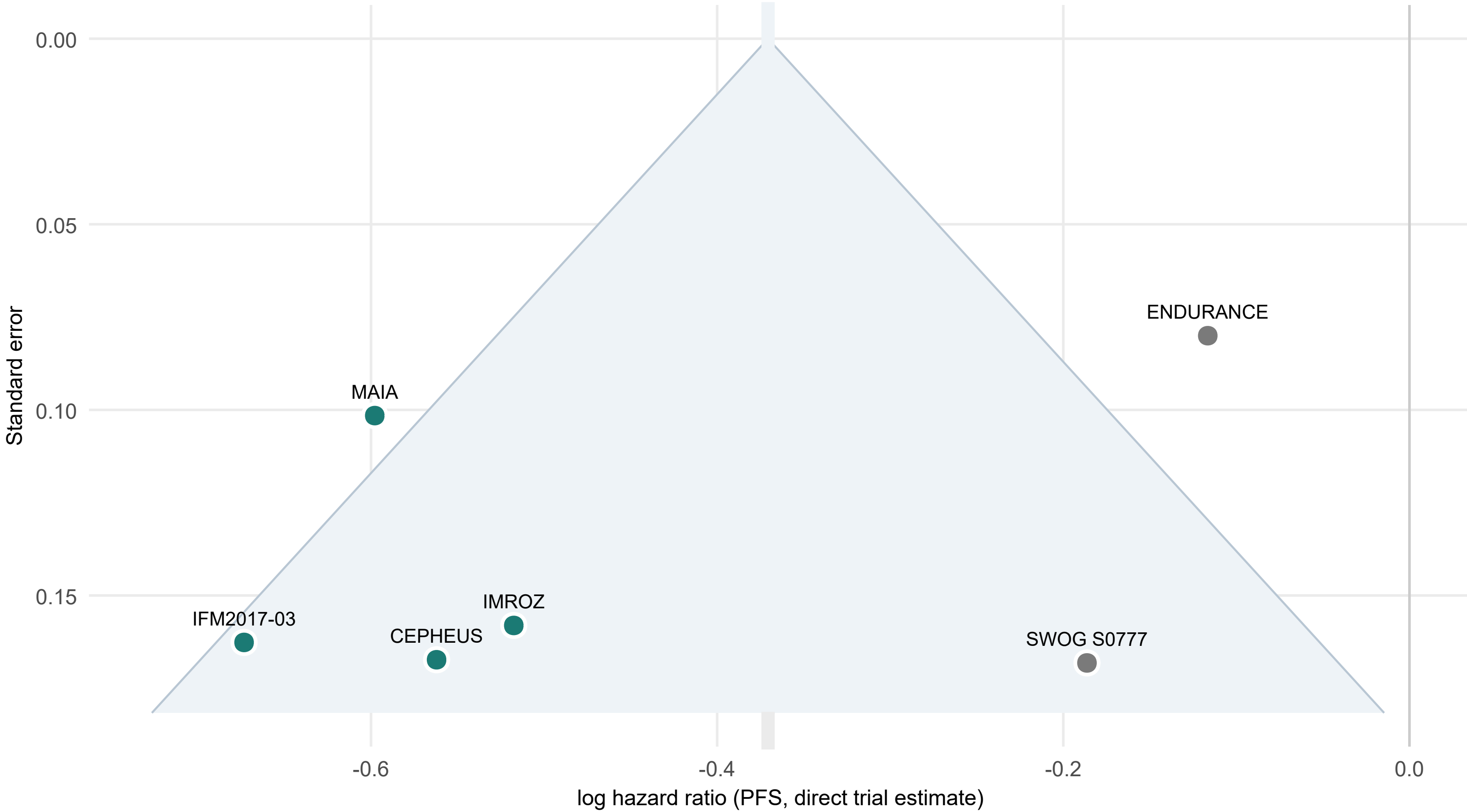

Visual inspection only. No formal small-study effect test (Egger's regression or equivalent) is reported because only 6 RCTs were available.

No pooled effect is shown.

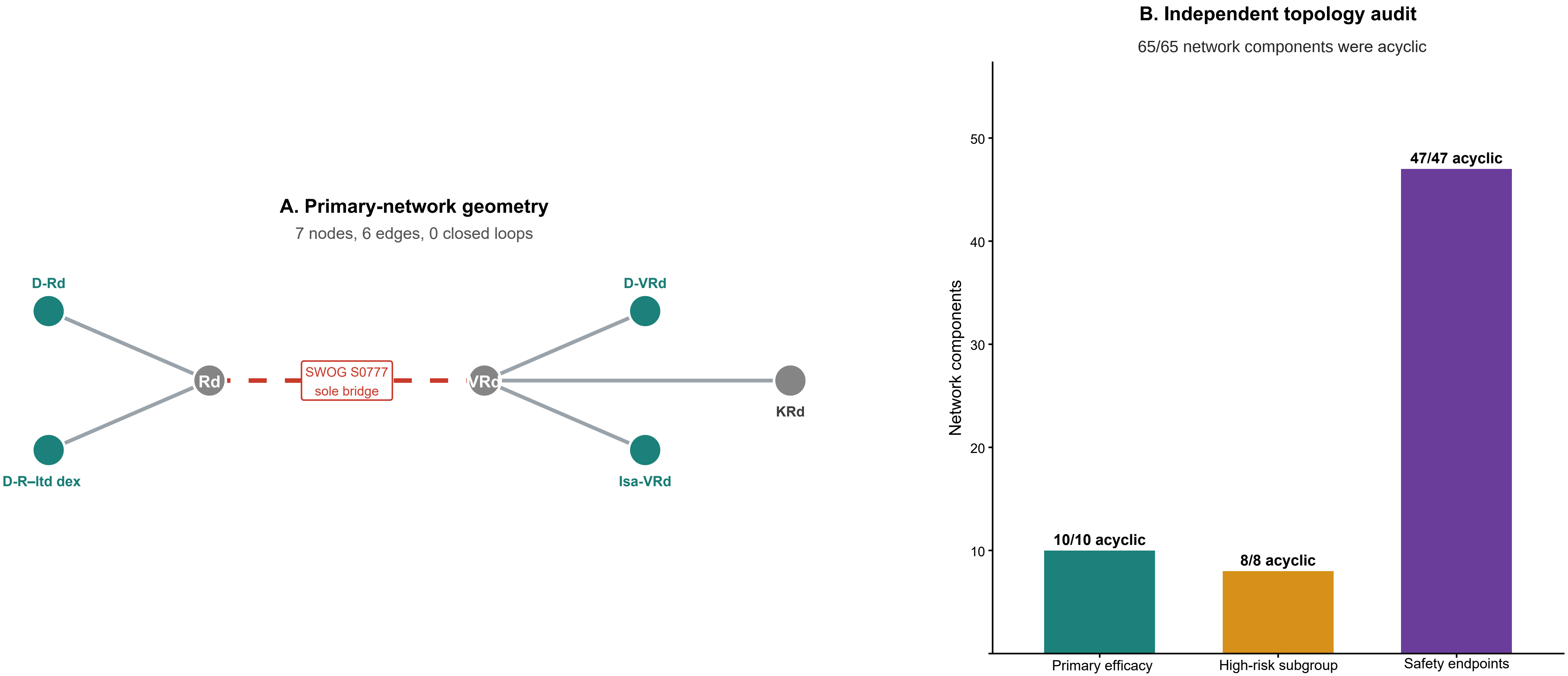

The primary network was acyclic (no closed loops); therefore, node-splitting and direct-versus-indirect inconsistency assessment were not possible.

eFigure 5. Rankograms: posterior probability of each rank

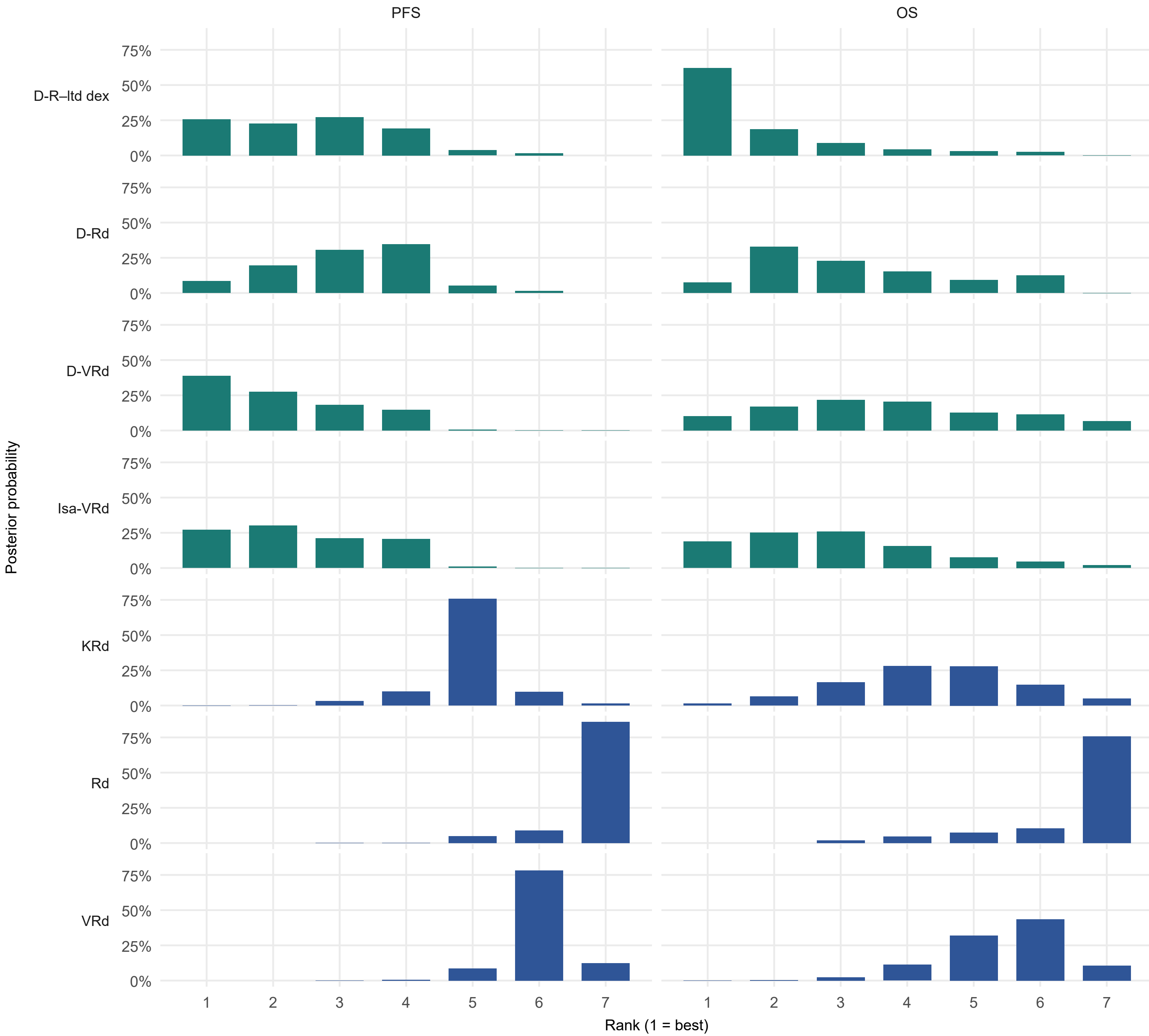

Common-effect Bayesian NMA.

eFigure 6A. Network meta-analysis relative effects versus Rd: PFS

Common-effect Bayesian NMA, ordered by SUCRA.

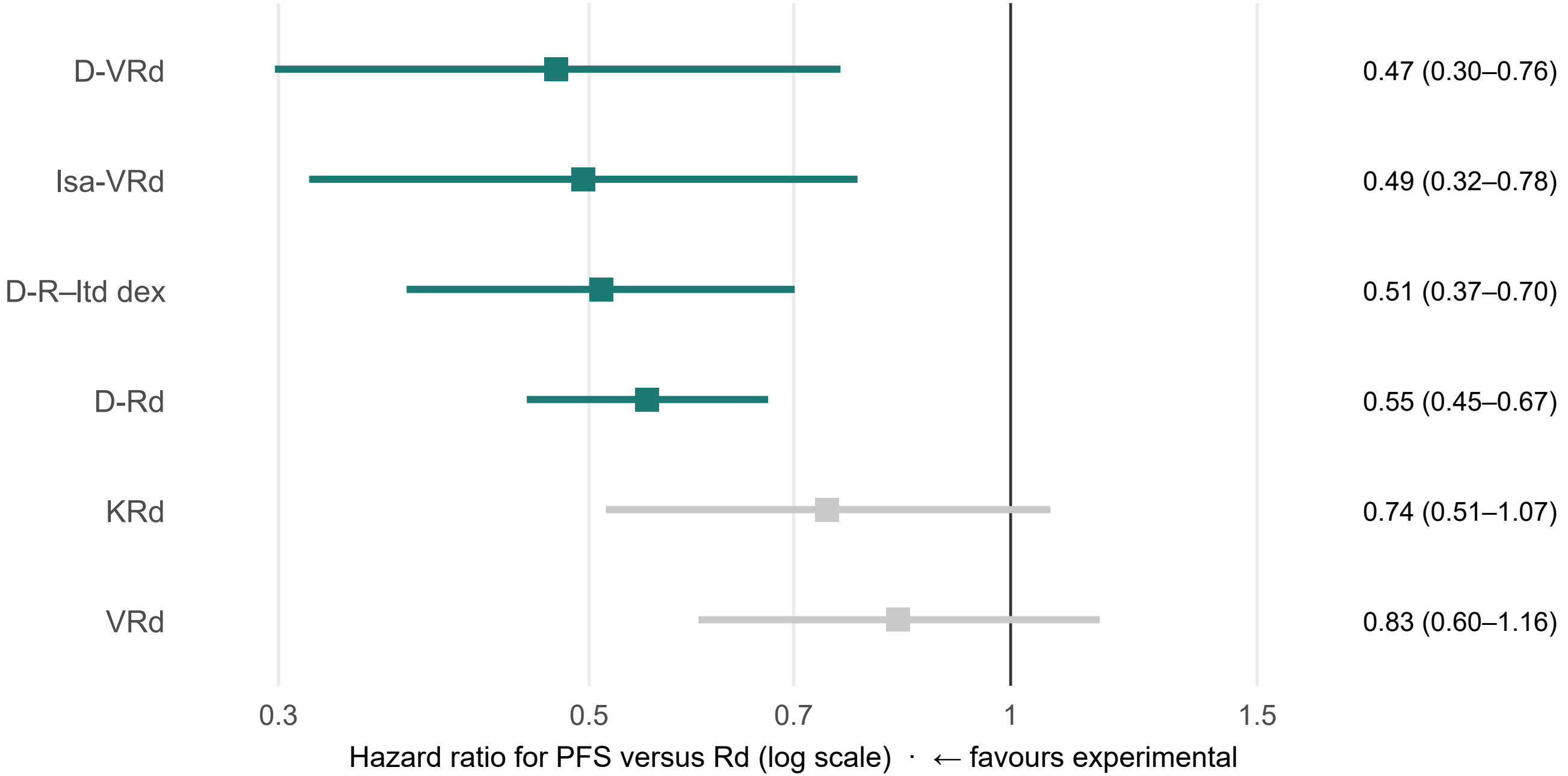

Solid intervals exclude 1; muted intervals include 1. Bayesian credible intervals are not significance tests. Teal = anti-CD38 regimen. Reference treatment: Rd. Because the contrasts share a common comparator these estimates are mutually comparable, unlike the trial-level direct estimates in Figures 3 and 4.

eFigure 6B. Network meta-analysis relative effects versus Rd: OS

Common-effect Bayesian NMA, ordered by SUCRA.

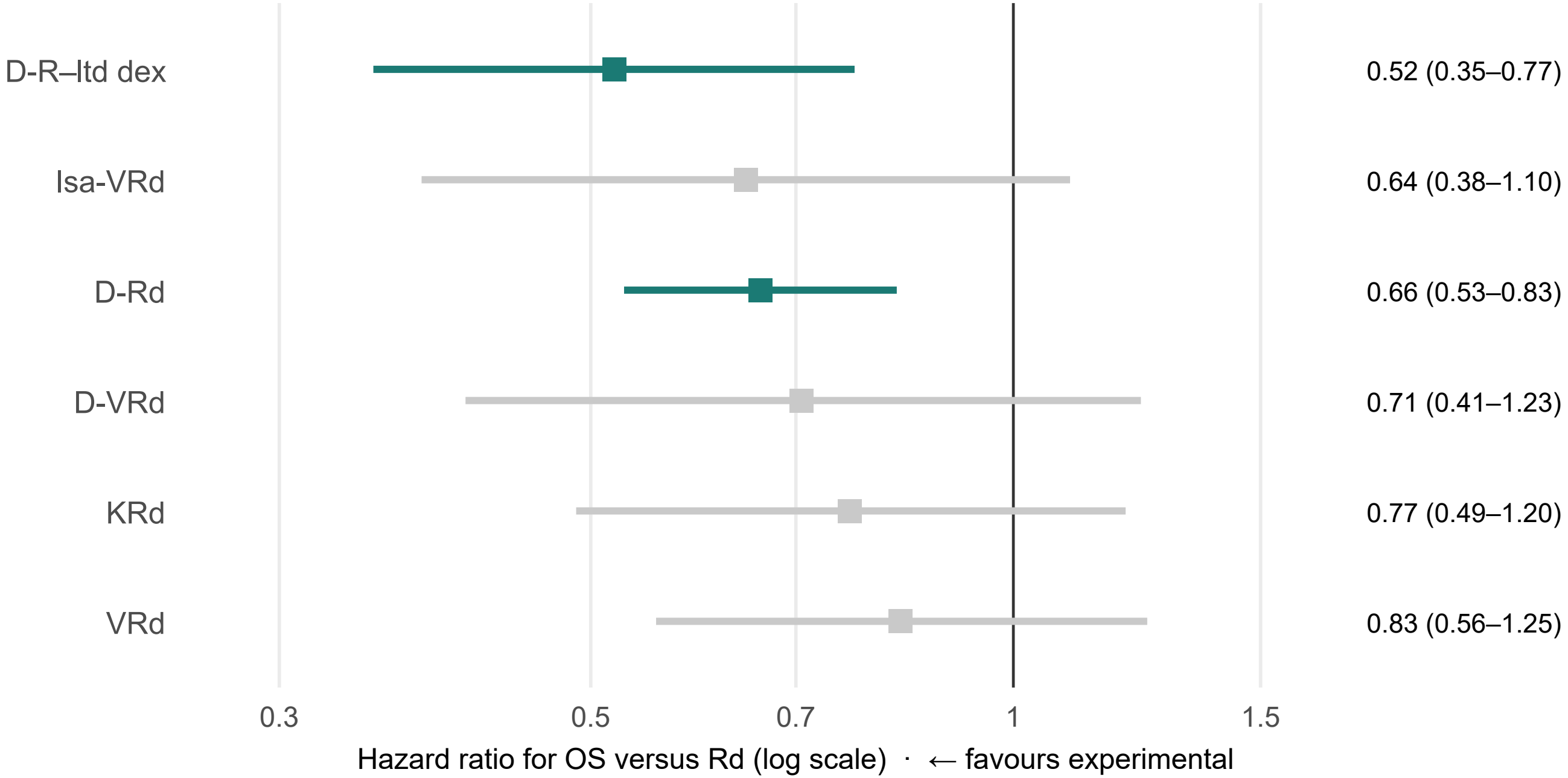

Solid intervals exclude 1; muted intervals include 1. Bayesian credible intervals are not significance tests. Teal = anti-CD38 regimen. Reference treatment: Rd. Because the contrasts share a common comparator these estimates are mutually comparable, unlike the trial-level direct estimates in Figures 3 and 4.

eFigure 7. Grade ≥3 adverse events by trial and randomised arm

	CEPHEUS D-VRd	CEPHEUS VRd	ENDURANCE KRd	ENDURANCE VRd	IFM2017-03 D-R–ltd dex	IFM2017-03 Rd	IMROZ Isa-VRd	IMROZ VRd	MAIA D-Rd	MAIA Rd
Anaemia	13% 26/197	12% 23/195			12% 24/200	3% 3/95	17% 46/263	16% 29/181	17% 62/364	22% 79/365
Cardiac failure			4% 19/526	1% 6/527						
Diarrhoea	12% 24/197	9% 18/195	3% 16/526	4% 23/527	4% 8/200	1% 1/95	8% 20/263	8% 15/181	9% 33/364	6% 22/365
Infection (overall)	40% 79/197	32% 62/195			19% 38/200	21% 20/95	45% 118/263	38% 69/181	43% 155/364	30% 108/365
Lung infection			5% 27/526	2% 9/527						
Neutropenia	44% 87/197	30% 58/195			55% 110/200	24% 23/95	54% 143/263	37% 67/181	54% 197/364	37% 135/365
Peripheral neuropathy					2% 3/200	1% 1/95				
Peripheral sensory neuropathy	8% 16/197	8% 16/195	1% 3/526	7% 39/527			7% 19/263	6% 11/181	2% 9/364	1% 2/365
Pneumonia	14% 28/197	13% 25/195			6% 12/200	9% 9/95	20% 53/263	13% 23/181	20% 71/364	11% 39/365
Thrombocytopenia	28% 56/197	20% 39/195			10% 19/200	5% 5/95	30% 79/263	28% 50/181		
Thromboembolic event			5% 26/526	2% 11/527						

Grade ≥3 (%): 10, 20, 30, 40, 50

Each cell shows the event rate for one randomised arm (events/total); values are not pooled across trials. Blank cells indicate that the event was not reported for that arm.

eFigure 8. Sensitivity Analysis: Extended Network (9 Treatment Nodes)

Two randomized trials—Real-MM and ALCYONE—extend the network through VMP-based nodes.

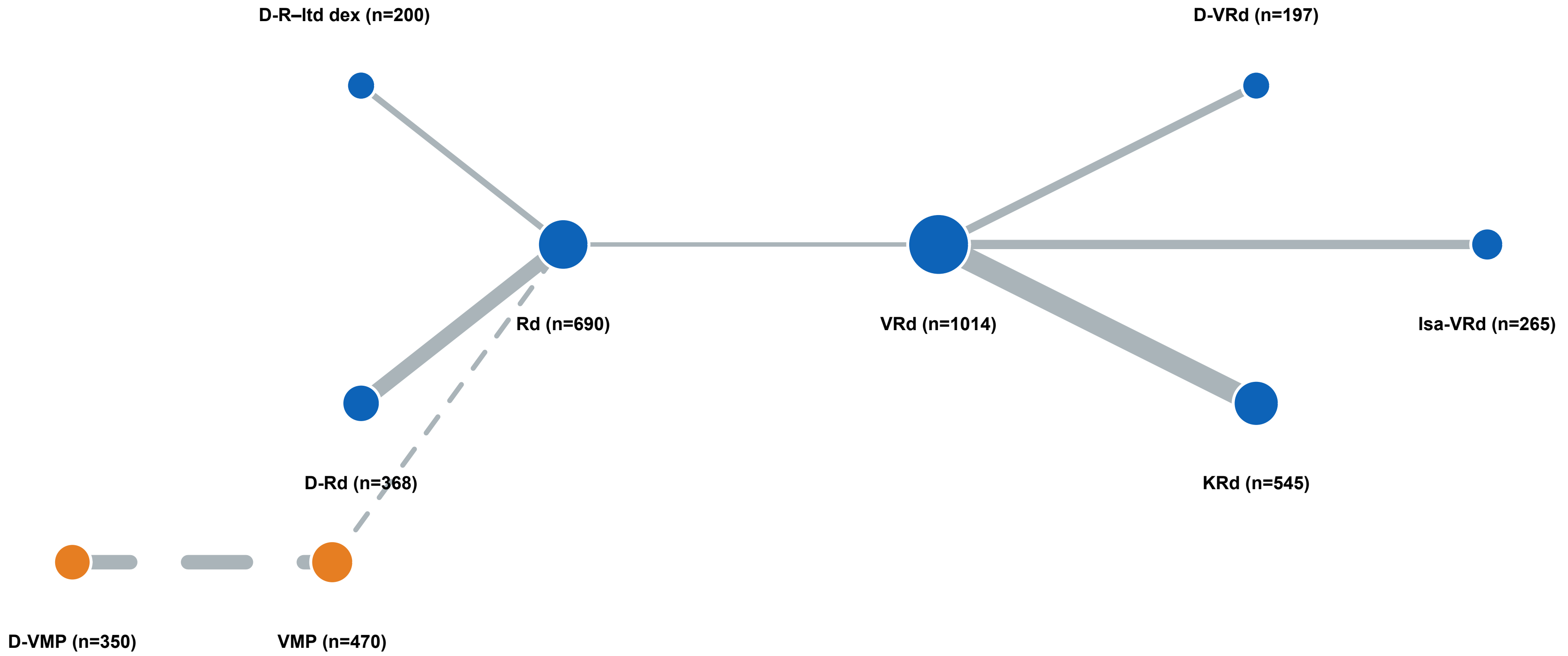

Real-MM is a randomized phase IV trial. The extended network remained acyclic; node-splitting was not estimable.

eFigure 9. High-risk cytogenetic subgroup: direct trial-level comparisons (PFS)

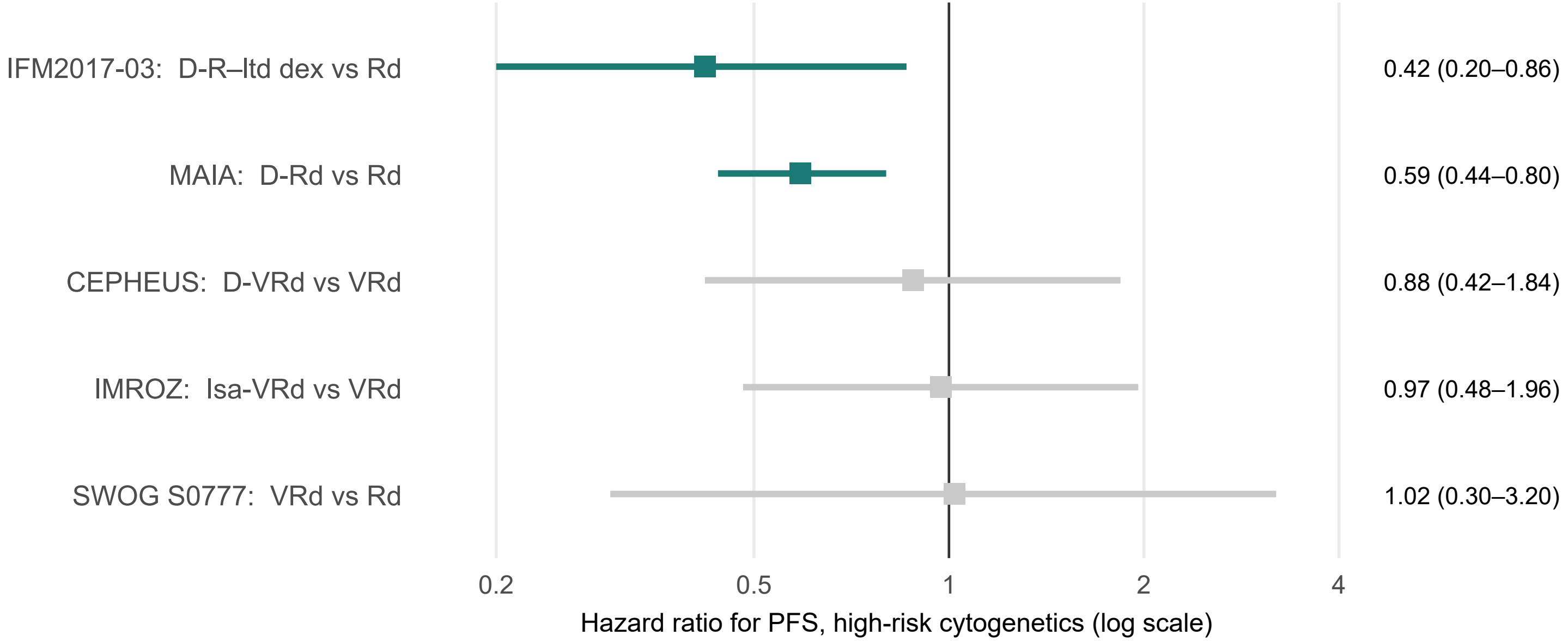

High risk = del(17p), t(4;14) and/or t(14;16). Solid intervals exclude 1; muted intervals include 1. Bayesian credible intervals are not significance tests. Subgroup sample sizes are small and these analyses are exploratory. For IFM2017-03 the high-risk count (n=31) comes from the per-protocol set, which excluded patients without evaluable cytogenetics; the full-analysis-set count (n=28) is the one reported in Table 3.

eFigure 10. Sensitivity Analysis: Ranking Stability (PFS SUCRA)

Alternative SWOG S0777 estimands

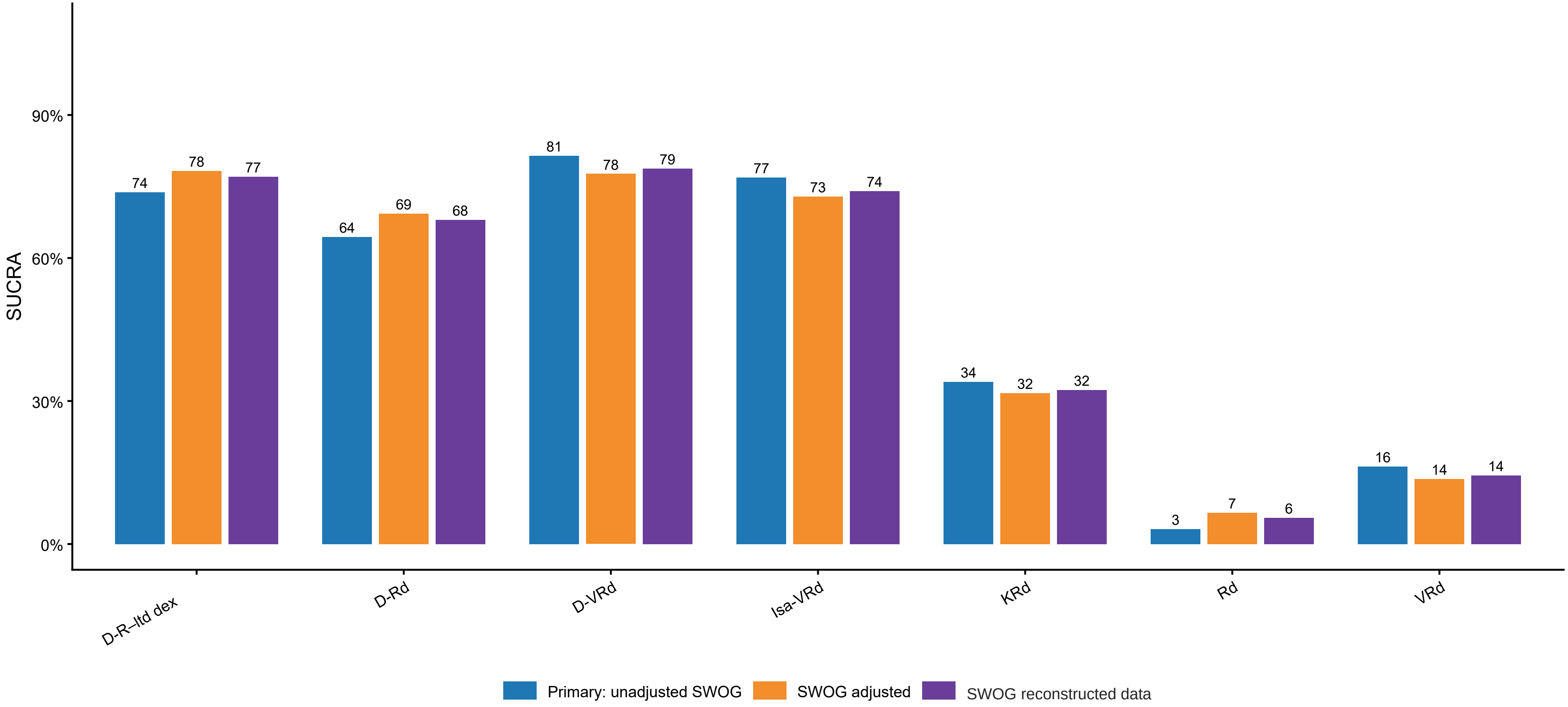

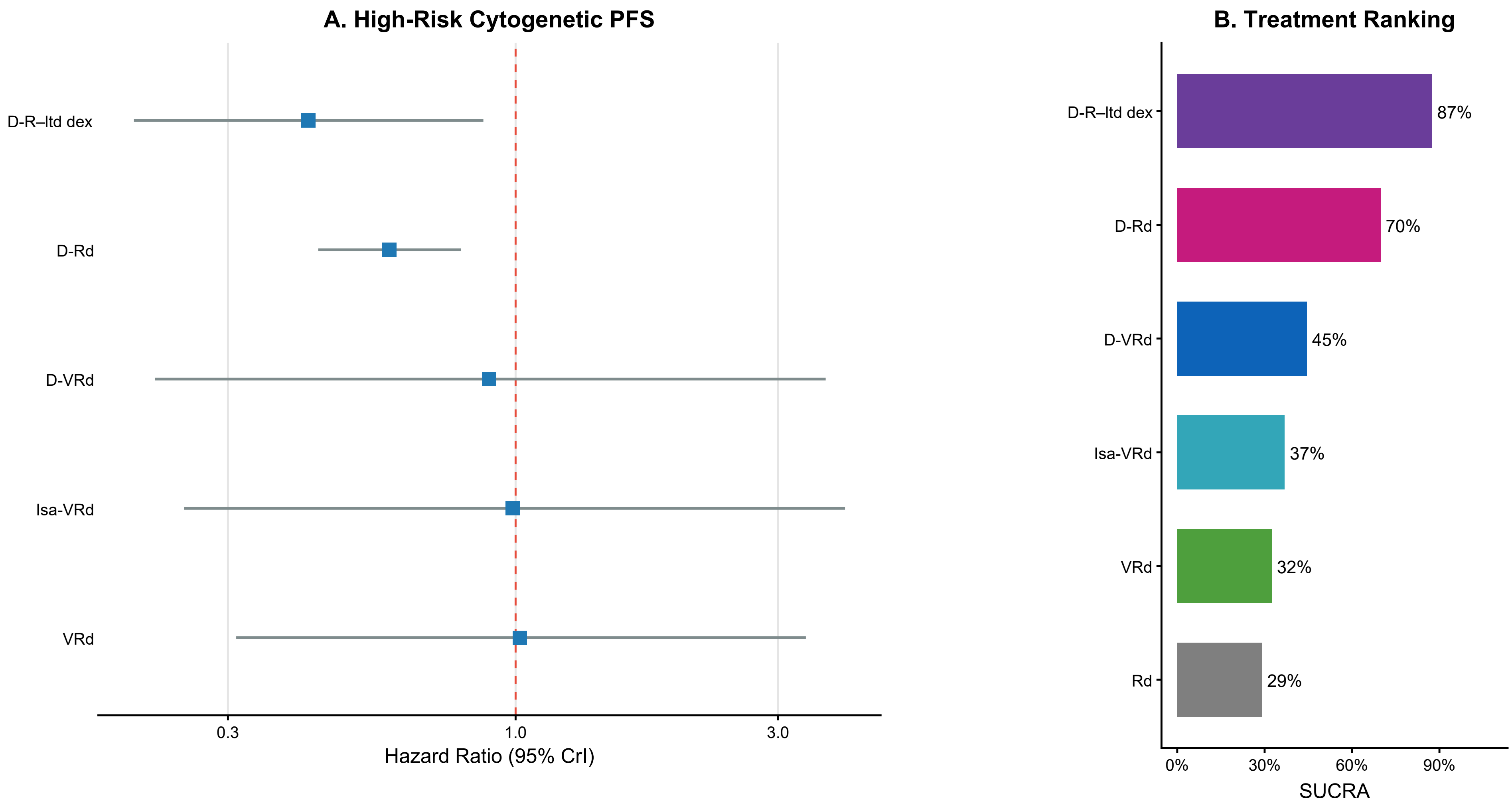
A. High-Risk Cytogenetic PFS
D-R–ltd dex
D-Rd
D-VRd
Isa-VRd
VRd
0.3
1.0
3.0
Hazard Ratio (95% CrI)
B. Treatment Ranking
D-R–ltd dex
D-Rd
D-VRd
Isa-VRd
VRd
Rd
87%
70%
45%
37%
32%
29%
0%
30%
60%
90%
SUCRA

eFigure 12. Forest plot of safety: grade ≥3 adverse events

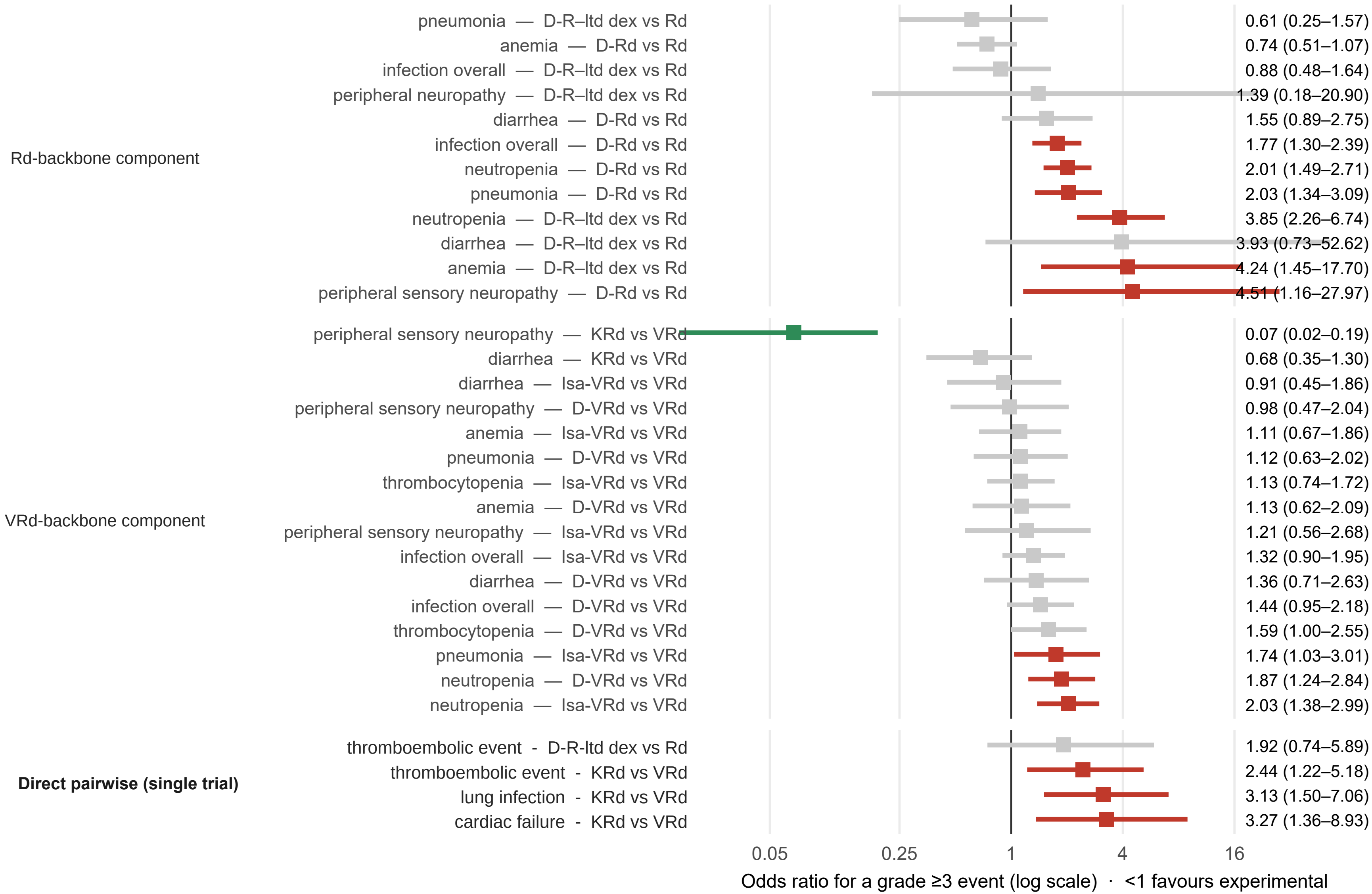

Connected comparisons were estimated by network meta-analysis; isolated comparisons are shown as direct pairwise estimates from a single trial. Red indicates higher odds with the experimental regimen, green lower odds, and grey estimates crossing 1.

eFigure 13. CEPHEUS data-version sensitivity: network estimates versus the primary analysis

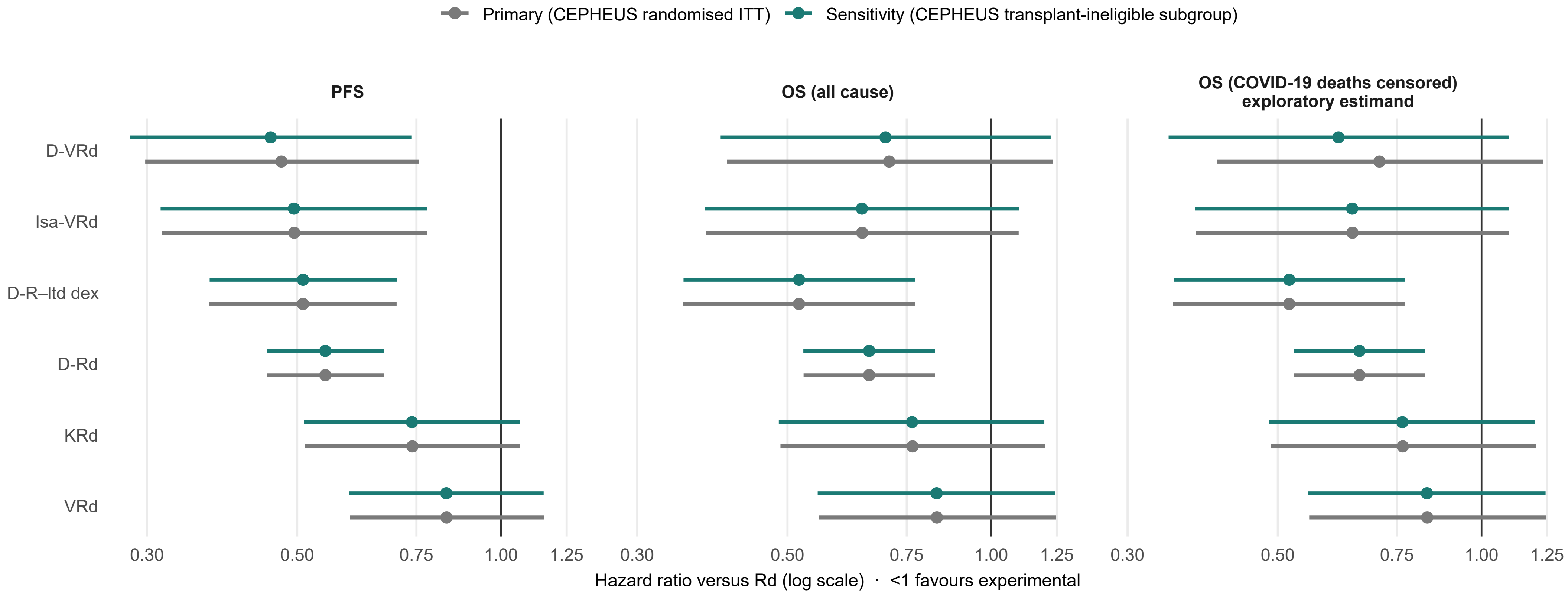

The peer-reviewed randomised ITT analysis of CEPHEUS (PFS HR 0.57, OS HR 0.85) remains the primary network input; the transplant-ineligible final subgroup is a sensitivity layer only, and only the CEPHEUS edge is replaced. Censoring COVID-19 deaths changes the estimand, so that panel is exploratory and never substitutes for all-cause overall survival. Any rank movement is reported as observed and is not suppressed.

eFigure 14. Random-effects prior sensitivity analysis

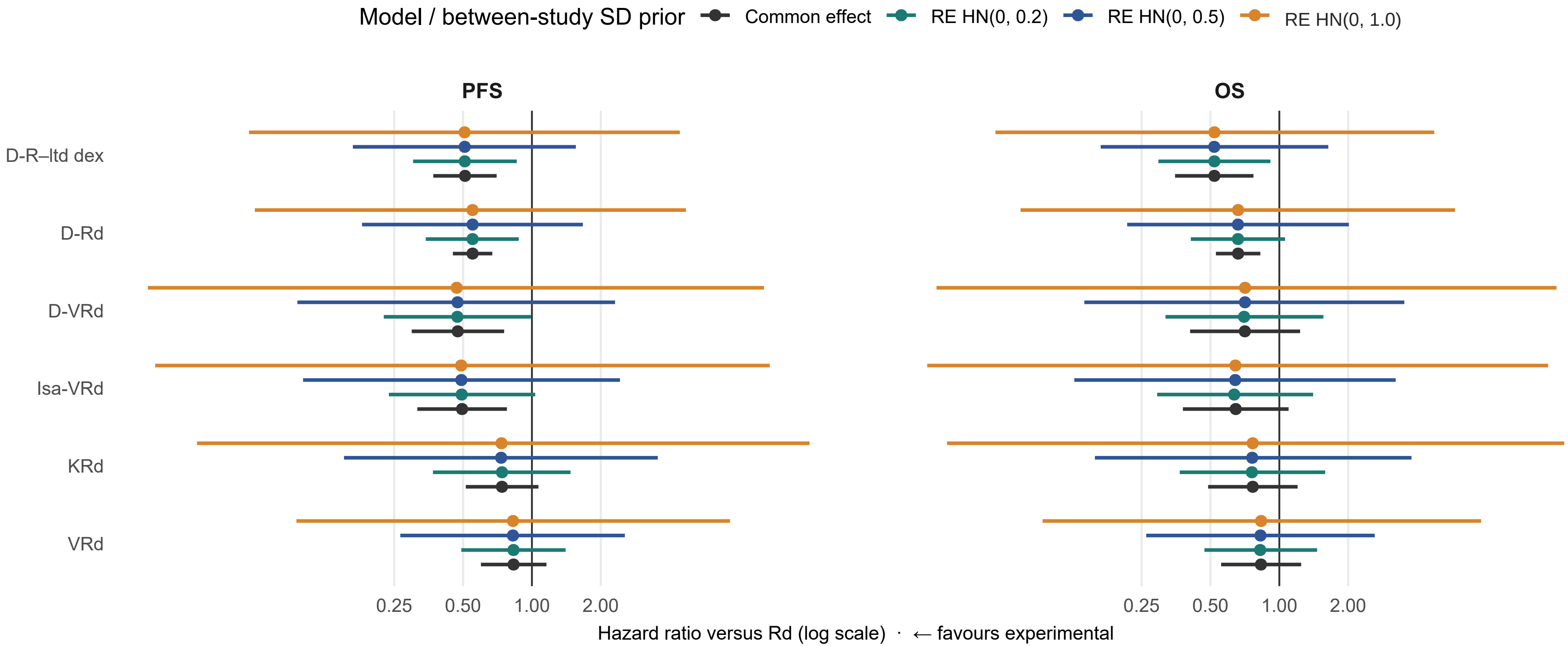

The primary analysis is a common-effect model, which the network geometry forces: every comparison is informed by a single trial, so the between-study standard deviation is not identified by the data and is driven entirely by its prior.
Half-normal priors of increasing scale are shown to make that dependence explicit. Point estimates are essentially unchanged; credible intervals widen as the prior admits more heterogeneity. Read this as a transparency analysis, not as a competing primary model.
